# Supplementary material for: THRONCAT: metabolic labeling of newly synthesized proteins using a bioorthogonal threonine analog
Source: Nat Commun. 2023 Jun 8;14:3367. doi: 10.1038/s41467-023-39063-7 (PMC10250548; doi:10.1038/s41467-023-39063-7)
Supplement: Supplementary file 1 — Supplementary Information [file 41467_2023_39063_MOESM1_ESM.docx]

**THRONCAT: Metabolic labeling of newly synthesized proteins using a bioorthogonal threonine analog**

Bob J. Ignacio^a^, Jelmer Dijkstra^b,d^, Natalia Mora^c,§^, Erik F.J. Slot^c,§^, Margot J. van Weijsten^a^, Erik Storkebaum^c,#^, Michiel Vermeulen^b,d,#^, Kimberly M. Bonger^*^

1. Department of Synthetic Organic Chemistry, Chemical Biology Lab, Radboud University, Heyendaalseweg 135, 6525AJ Nijmegen, the Netherlands
2. Department of Molecular Biology, Radboud Institute for Molecular Life Sciences, Oncode Institute, Radboud University, Nijmegen, the Netherlands
3. Molecular Neurobiology Laboratory, Donders Center for Neuroscience, Donders Institute for Brain, Cognition and Behaviour and Faculty of Science, Radboud University, Nijmegen, the Netherlands
4. Division of Molecular Genetics, The Netherlands Cancer Institute, Amsterdam, The Netherlands

^§^These authors equally contributed to this work.

^#^ These authors equally contributed to this work.

*Correspondence to Kimberly M. Bonger: k.bonger@science.ru.nl

**Supporting Information**

**Table of contents**

**Supplementary Figures ……………………………………………………………………….S2**

**Supplementary Table 1……………………………………………………………………….S25**

**Supplementary Table 2……………………………………………………………………….S26**

**Supplementary Discussion 1 ….……………………………………………………………S27**

**Supplementary Note 1 ……………………………………………..…………………………S28**

**Supplementary References .………………………………………………………………..S36**

# **Supplementary Figures**


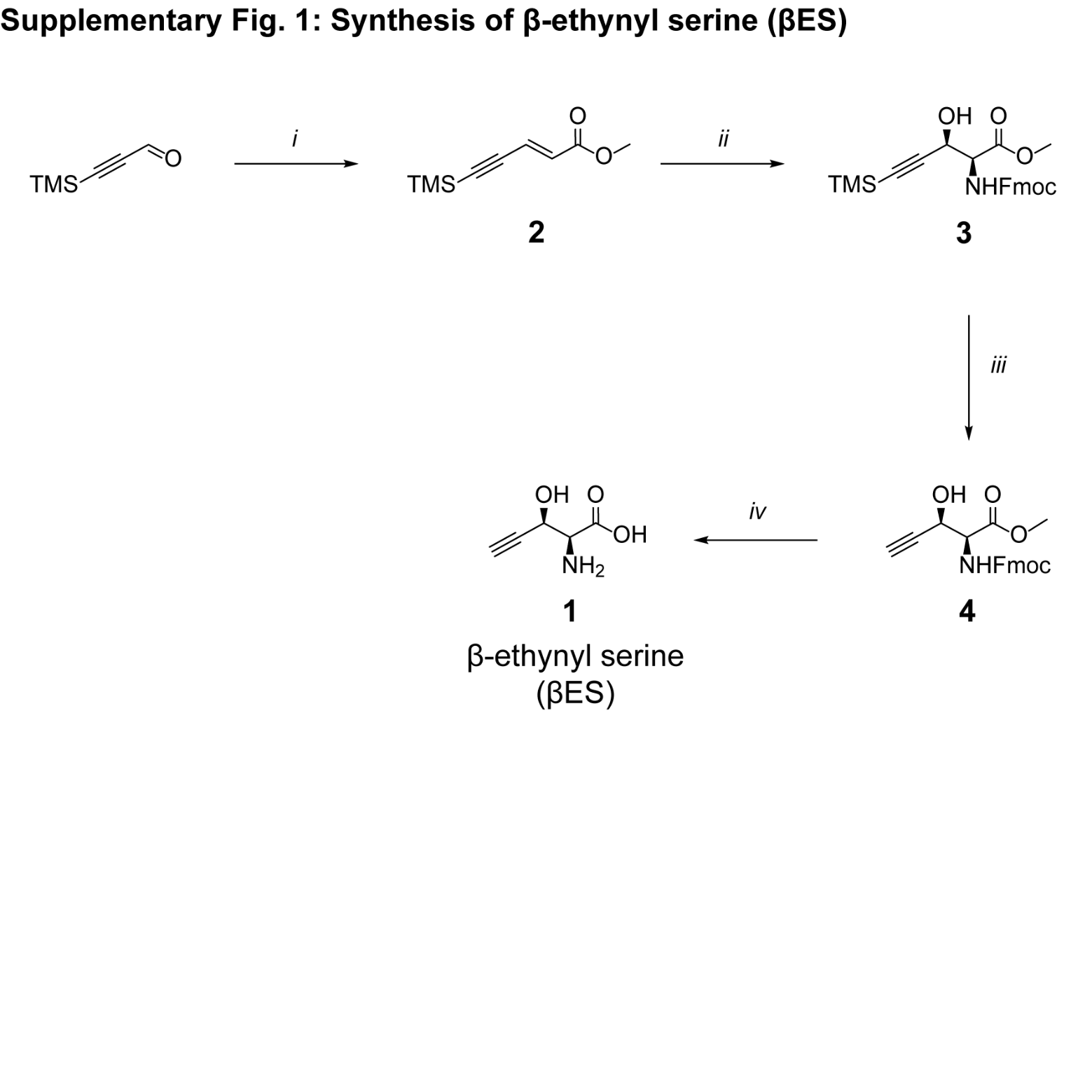


**Supplementary Figure 1:** **Synthesis of (2S,3R)-2-amino-3-hydroxypent-4-ynoic acid (β-ethynylserine, 1).** Reagents and yields: i) methyl (triphenylphosphoranylidene)acetate, THF, 95%; ii) (DHQD)_2_AQN, K_2_OsO_4_ · 2 H_2_O, NaOH, 5, H_2_O/n-PrOH, 31%; iii) TBAF, DCM, 82%; iv) LiOH, H_2_O/MeCN, 93% (> 98% de, 86% ee). See Supplementary Note 1 for full synthetic details.


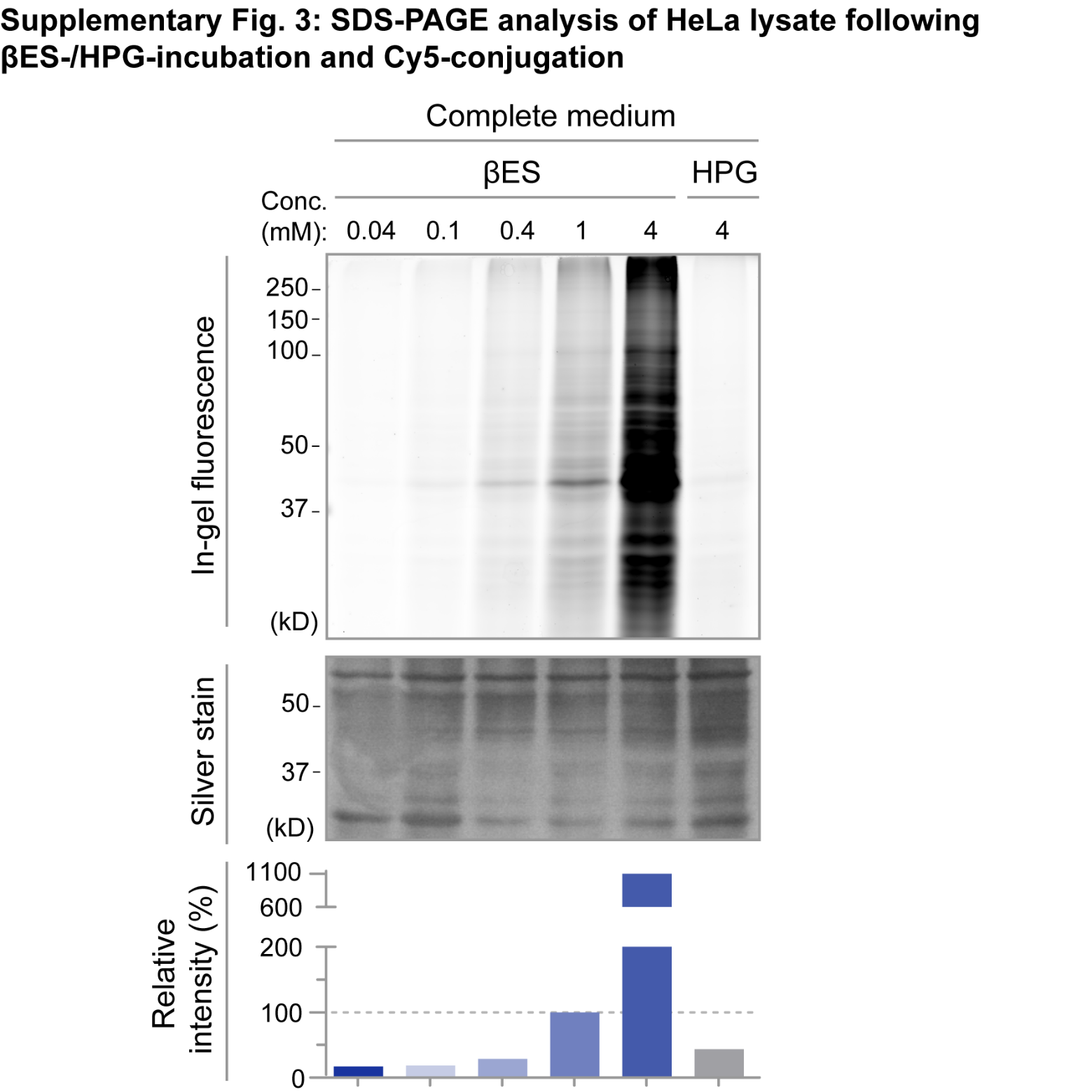


**Supplementary Figure 2: In-gel visualization of βES or HPG incorporation into the HeLa proteome**. HeLa cells were incubated for 1 h with the indicated concentrations of βES or HPG in LB medium. HeLa cell lysate was conjugated to Cy5-azide and visualized by in-gel fluorescence after SDS-PAGE separation. Silver stain panel shows total protein in lysates. Bar chart shows relative intensity of in-gel fluorescence normalized to silver stain intensity. Relative intensity bar chart is normalized to ‘βES 1 mM’ signal, which is set to 100%. The experiment was performed in biological duplicate with similar results. βES, β-ethynyl serine; HPG, homopropargylglycine.

­
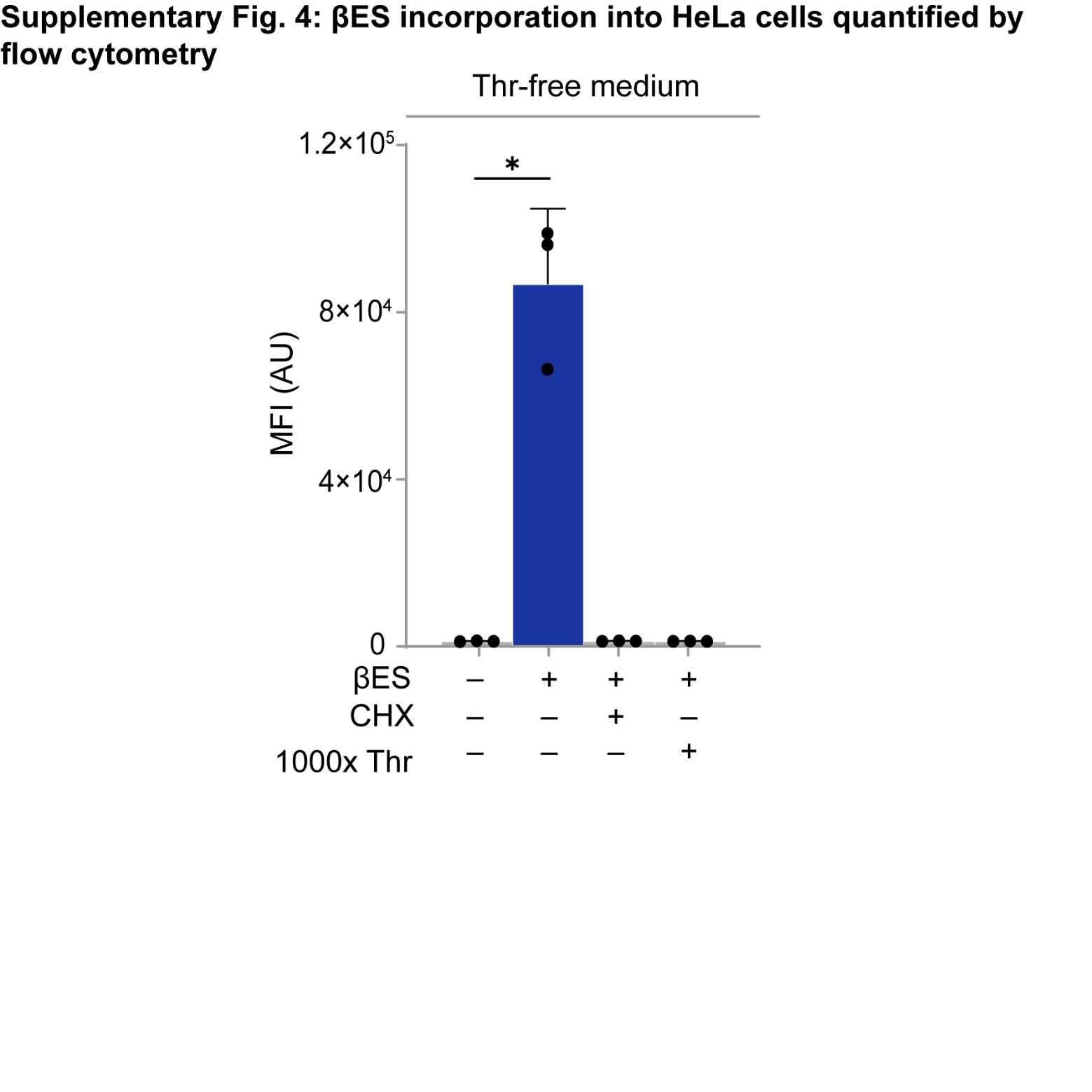


**Supplementary Figure 3: Flow cytometry quantification of βES incorporation into the HeLa proteome**. HeLa cells were starved for 1 h in threonine-free medium and incubated for 1 h with 1 μM βES in threonine-free medium. Control cells were left untreated or treated with 1 μM βES and the protein synthesis inhibitor cycloheximide (CHX) or, 1 μM βES and an excess of threonine. Incorporated βES was conjugated to Cy5-azide for quantification. MFI, Mean fluorescence intensity; AU, arbitrary units; Thr, threonine. **P =* 0.0146 determined by an unpaired two-tailed Students t test. Error bars represent s.d. Sample size is *n* = 3.


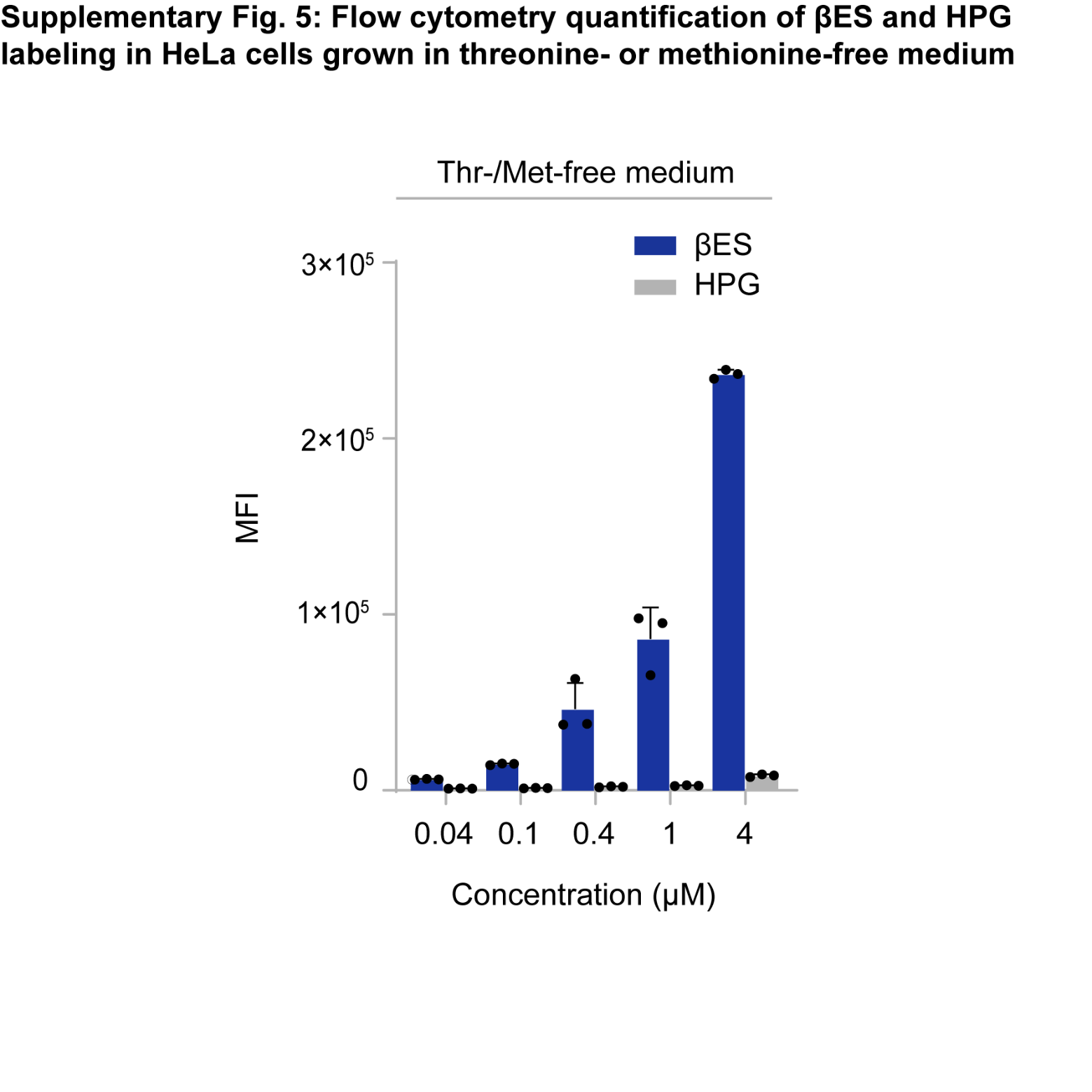


**Supplementary Figure 4: Flow cytometry quantification of βES and HPG incorporation into the HeLa proteome** **in threonine- or methionine-free medium.** HeLa cells were starved for 1 h in threonine-free or methionine-free medium and incubated for 1 h with the indicated concentrations of βES in threonine-free medium or HPG in methionine-free medium, respectively. Incorporated analog was conjugated to Cy5-azide for quantification. MFI, mean fluorescence intensity; AU, arbitrary units; Thr, threonine; Met, methionine. Error bars represent s.d. Sample size is *n* = 3.

**
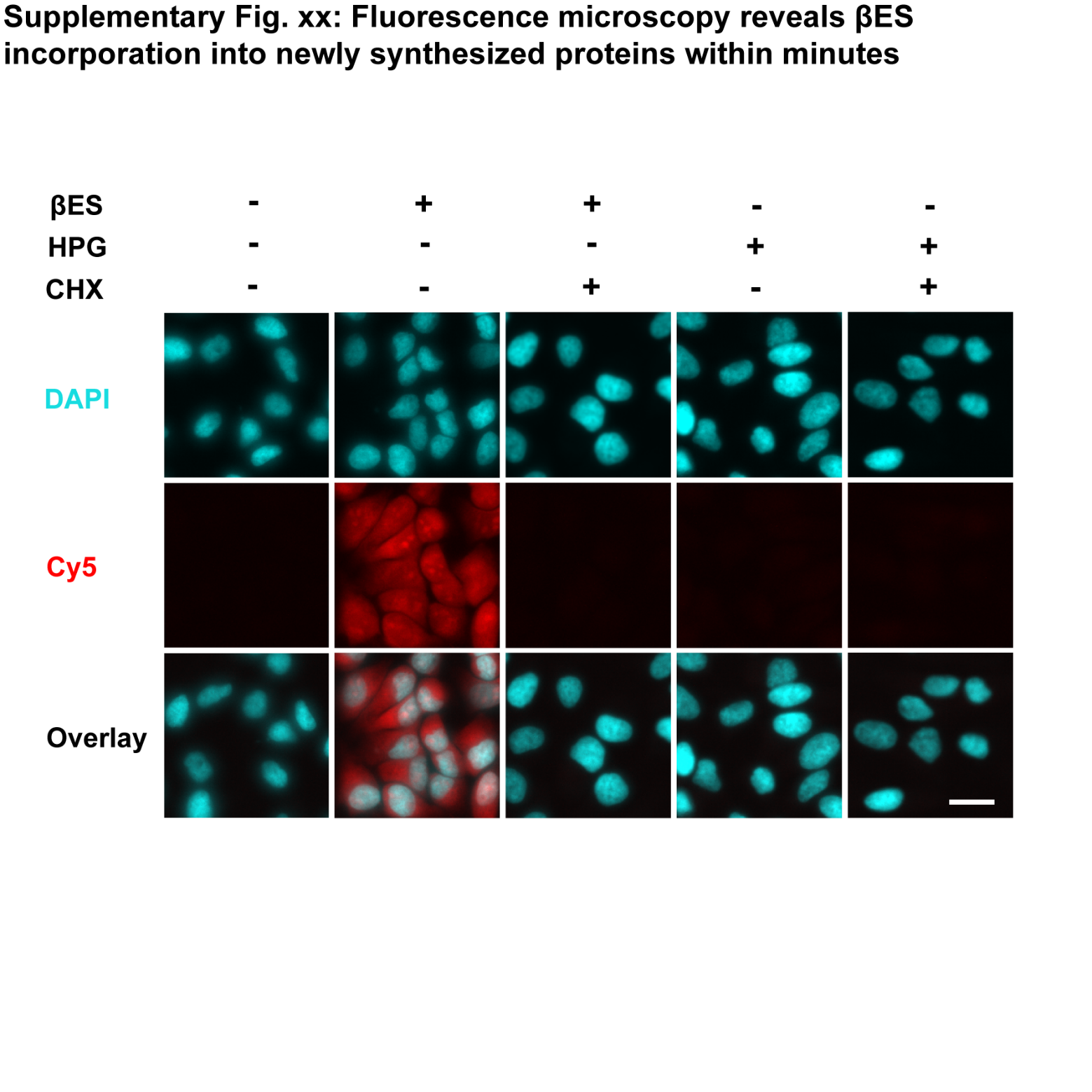
**

**Supplementary Figure 5: Fluorescence microscopy of NSPs in HeLa cells using THRONCAT.** Representative fluorescent images of HeLa cells treated for 10 min with 4 mM βES or 4 mM HPG in complete medium, with or without 100 μM cycloheximide (CHX), followed by conjugation to Cy5-azide (red). HeLa nuclei are stained with DAPI (cyan). The experiments were repeated twice independently with similar results. Representative images are shown here. Scale bar, 20 μm. βES, β-ethynyl serine; HPG, homopropargylglycine; DAPI, 4’,6-diamidino-2-phenylindole.


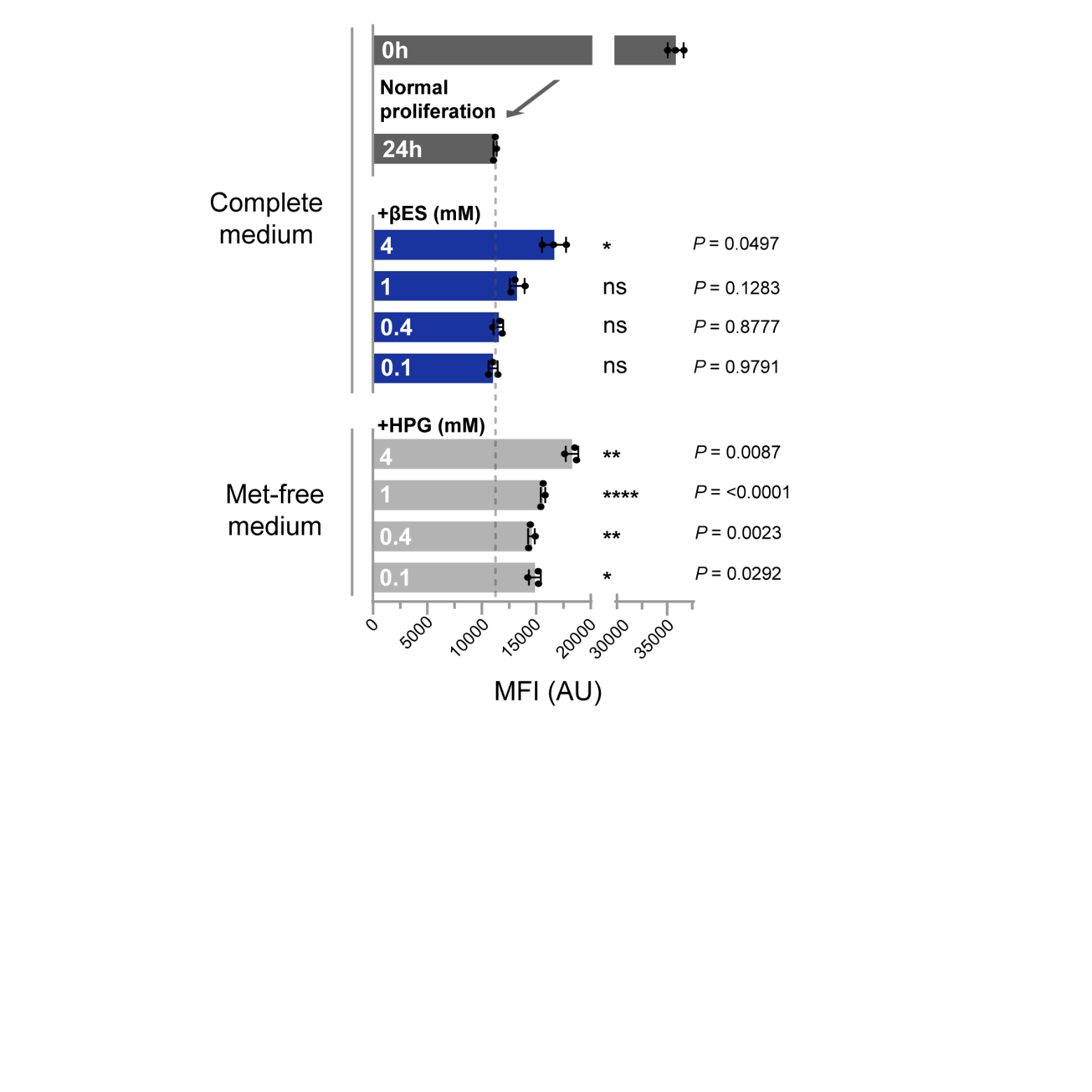


**Supplementary Figure 6:** **Flow cytometry proliferation assay of HeLa cells.** HeLa cells were labeled with Celltrace Violet and incubated without analog (control) or with indicated concentrations of analog for 24 h. Black arrow indicates the decrease in CellTrace fluorescence in control cells. Dotted line indicates the final fluorescent signal obtained from control cells. Significance determined by one-way Brown-Forsythe ANOVA, comparing treated samples with control (24h). Dunnett’s T3 multiple comparisons test was used to correct for multiple testing. Exact p-values and p-value summaries are given in the figure. Briefly, from top to bottom, ^*^*P* = 0.0497, *P* = 0.1283, *P* = 0.8777, *P* = 0.9791, ^**^*P* = 0.0087, ^****^*P* < 0.0001, ^**^*P* = 0.0023, ^*^*P* = 0.0292. Data are presented as mean values, error bars represent s.d. Sample size is n = 3. ns, not significant.

­­
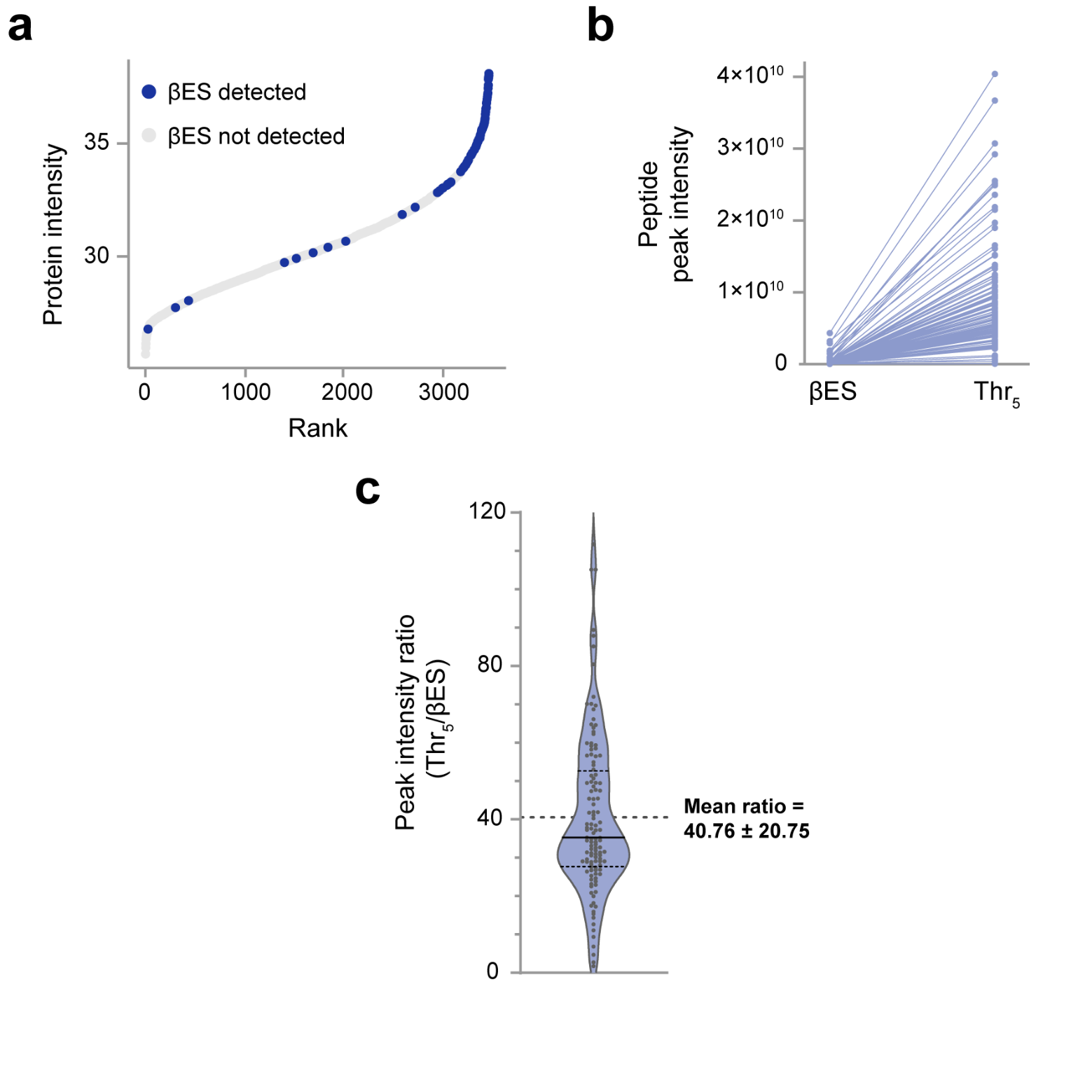


**Supplementary Figure 7: Pairwise comparison between peak intensities of βES-/Thr_5_-modified peptides. a**, Rank plot showing intensities for 3463 identified proteins. On average, we detected 142 peptides with a βES-modification, 9944 with a Thr_5_-modification and 7410 with unmodified threonine. Of the 142 peptides with a βES modification, which were derived from 90 abundant proteins (blue), 124 were also detected with a Thr_5_ modification. βES was not detected in 3373 proteins (gray). **b**, Dot plot showing peak intensities from LC-MS/MS spectra for 124 paired βES- or Thr_5_-modified peptides. Paired peptides are visualized with a connecting line. **c**, Violin plot showing ratios of peak intensities between paired βES- or Thr_5_-modified peptides. The mean peak intensity ratio (Thr_5_/βES) of the paired peptides is 40.76 and the standard deviation is 20.75. The median peak intensity ratio (Thr_5_/βES) of the paired peptides is 35.25, the 25% percentile is 27.71 and the 75% percentile is 52.65. Violin plot was created with Prism 9 (Graphpad) using medium smoothing. Statistics were performed with Prism 9. βES, β-ethynyl serine; Thr_5_, ^13^C_4_,^15^N-threonine.


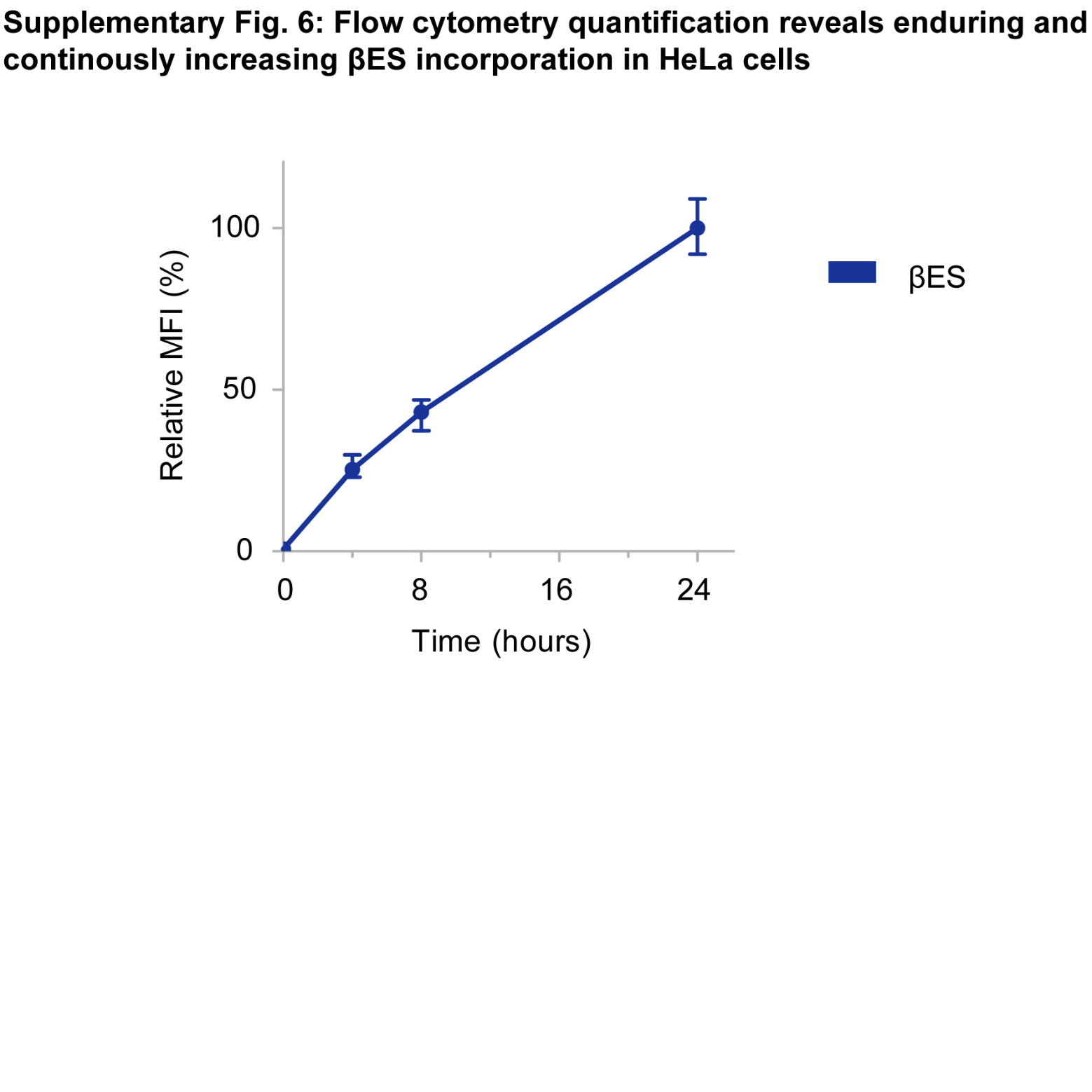


**Supplementary Figure 8: Flow cytometry quantification of βES incorporation into the HeLa proteome over time.** HeLa cells were incubated with 4 mM βES in complete medium for the indicated duration. Untreated HeLa cells were taken along as control (0 h). Incorporated analog was conjugated to Cy5-azide for quantification. Signals are normalized to that at 24 h, which is set to 100%. MFI, Mean fluorescence intensity. Error bars represent s.d. Sample size is *n* = 3.


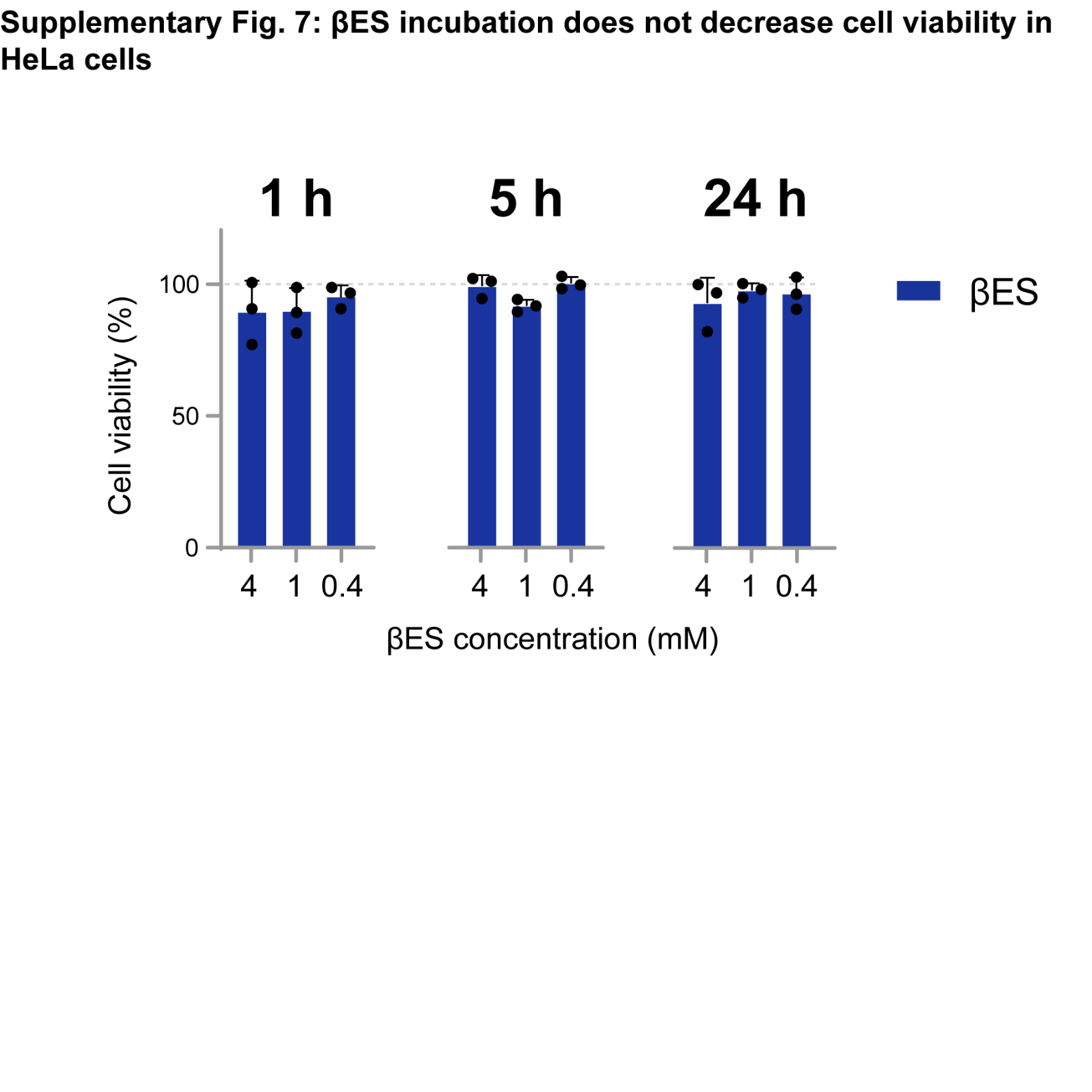


**Supplementary Figure 9: βES incubation does not decrease cell viability of HeLa cells.** HeLa cells were incubated with the indicated concentrations of βES in complete medium for the indicated duration. Cell viability was determined by exclusion of propidium iodide dye. Untreated HeLa cells were taken along as control. Signals are normalized to that of control, which is set to 100%. Data are presented as mean values, error bars represent s.d. Sample size is *n* = 3.


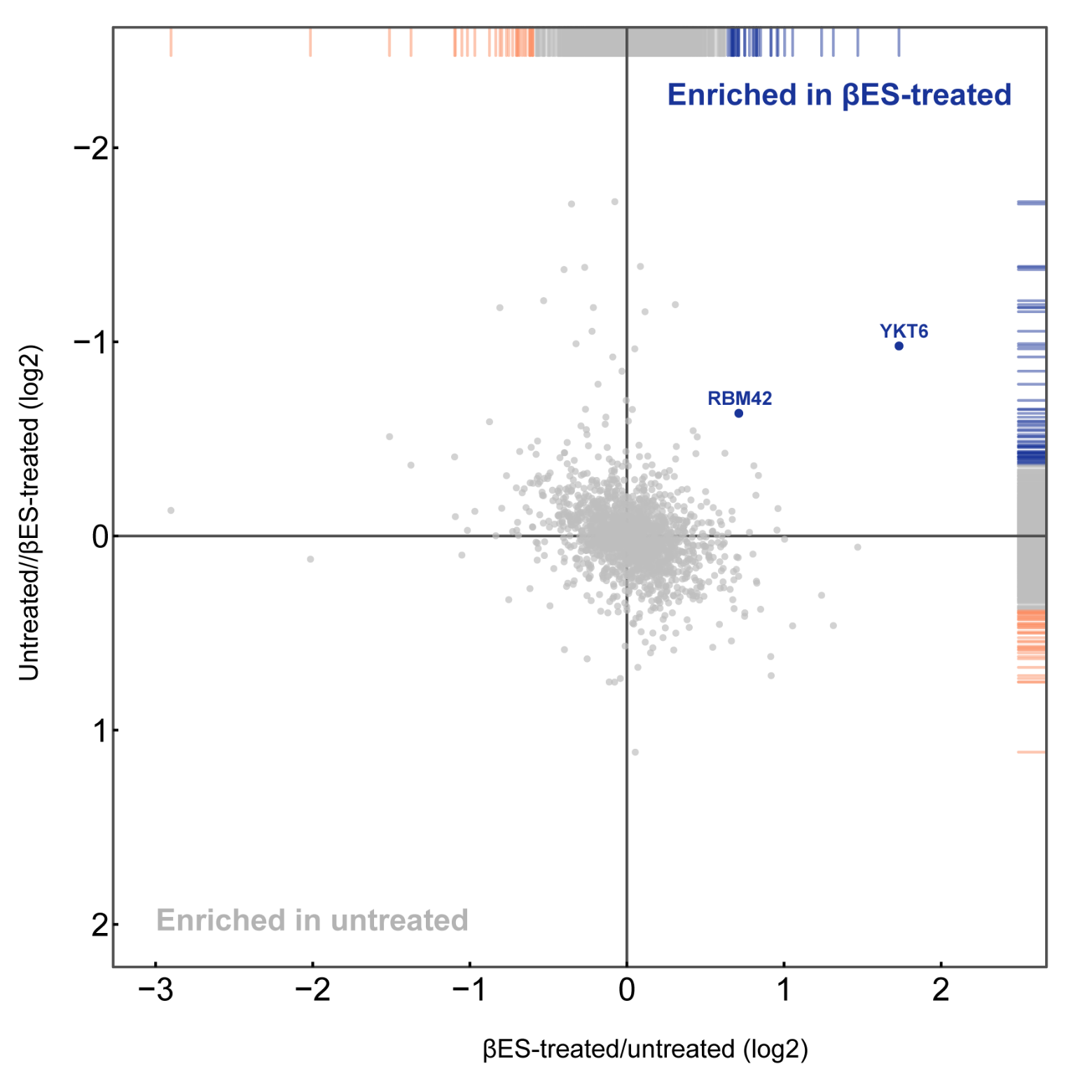


**Supplementary Figure 10: βES labeling does not induce a proteomic stress response in HeLa cells.** HeLa cells were incubated for 5 h in complete growth medium with or without 1 mM βES and whole cell lysates were subjected to LC-MS/MS analysis. Forward and reverse dimethyl labeling was used to determine differentially expressed proteins between βES-treated and untreated conditions. βES labeling did not induce a stress response and did not significantly change protein expression levels, except for those of RNA-binding protein 42 (RBM42) and Synaptobrevin homolog YKT6 (YKT6). This experiment was performed once without biological replicates (*n = 1*).

­

**
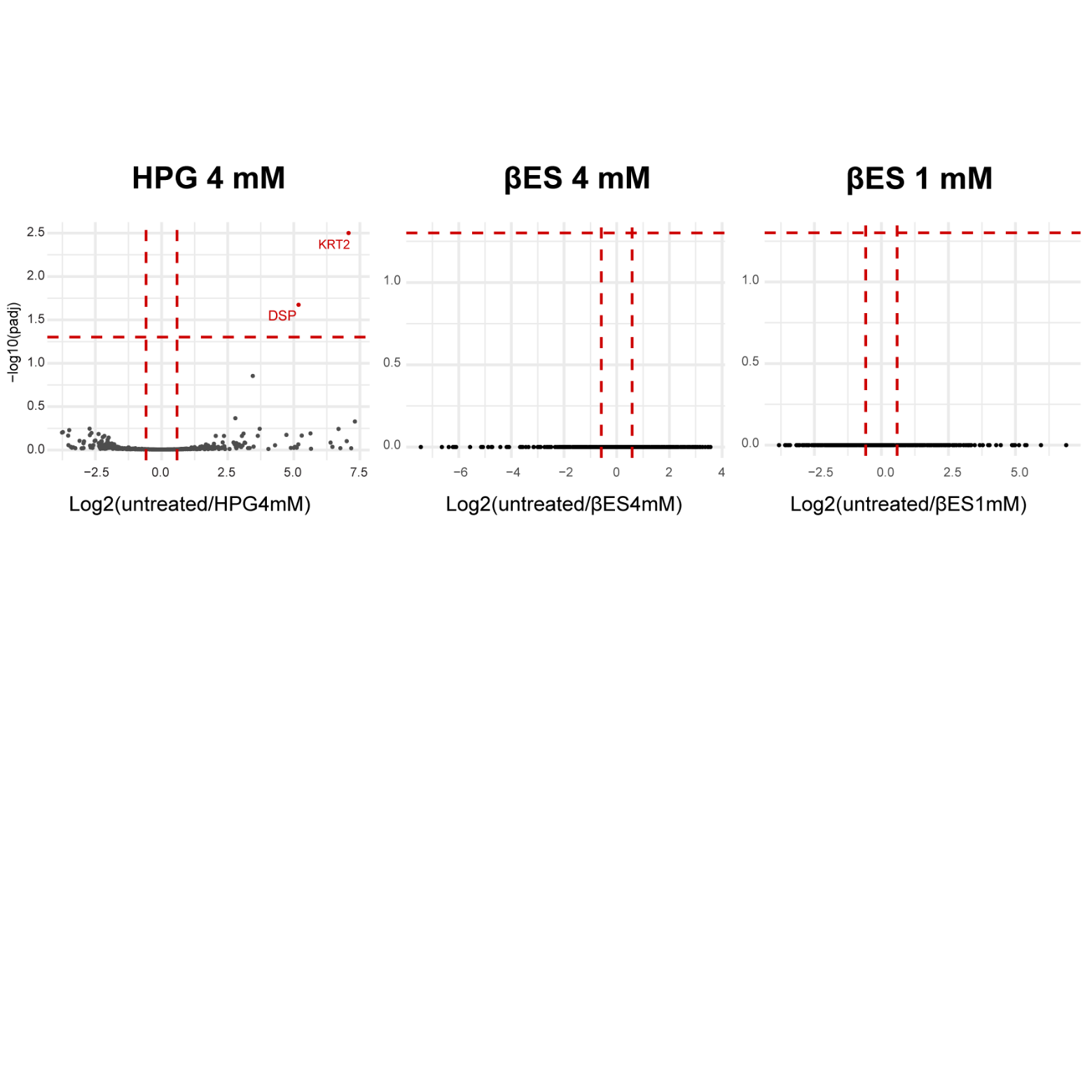
**

**Supplementary Figure 11: Volcano plots showing differential protein enrichment between untreated and βES-/HPG-treated HeLa cells.** HeLa cells were incubated with 1 mM or 4 mM βES in complete medium, 4 mM HPG in methionine-free medium or left untreated. After cell lysis and enrichment of newly synthesized proteins, digested peptides were subjected to LC-MS/MS analysis. Volcano plots show differential protein expression between the untreated condition and βES-/HPG-treated conditions for proteins found in both the untreated condition and treated conditions. Proteins with a FC > 1.5 and p.adj < 0.05 were considered to be significantly enriched in the treated conditions and all other proteins are considered to be background binders. Keratin (KRT2) and Desmoplakin (DSP), indicated as red dots, were the only two proteins found in the untreated condition that were significantly enriched in a treated condition and were therefor not considered to be background proteins.


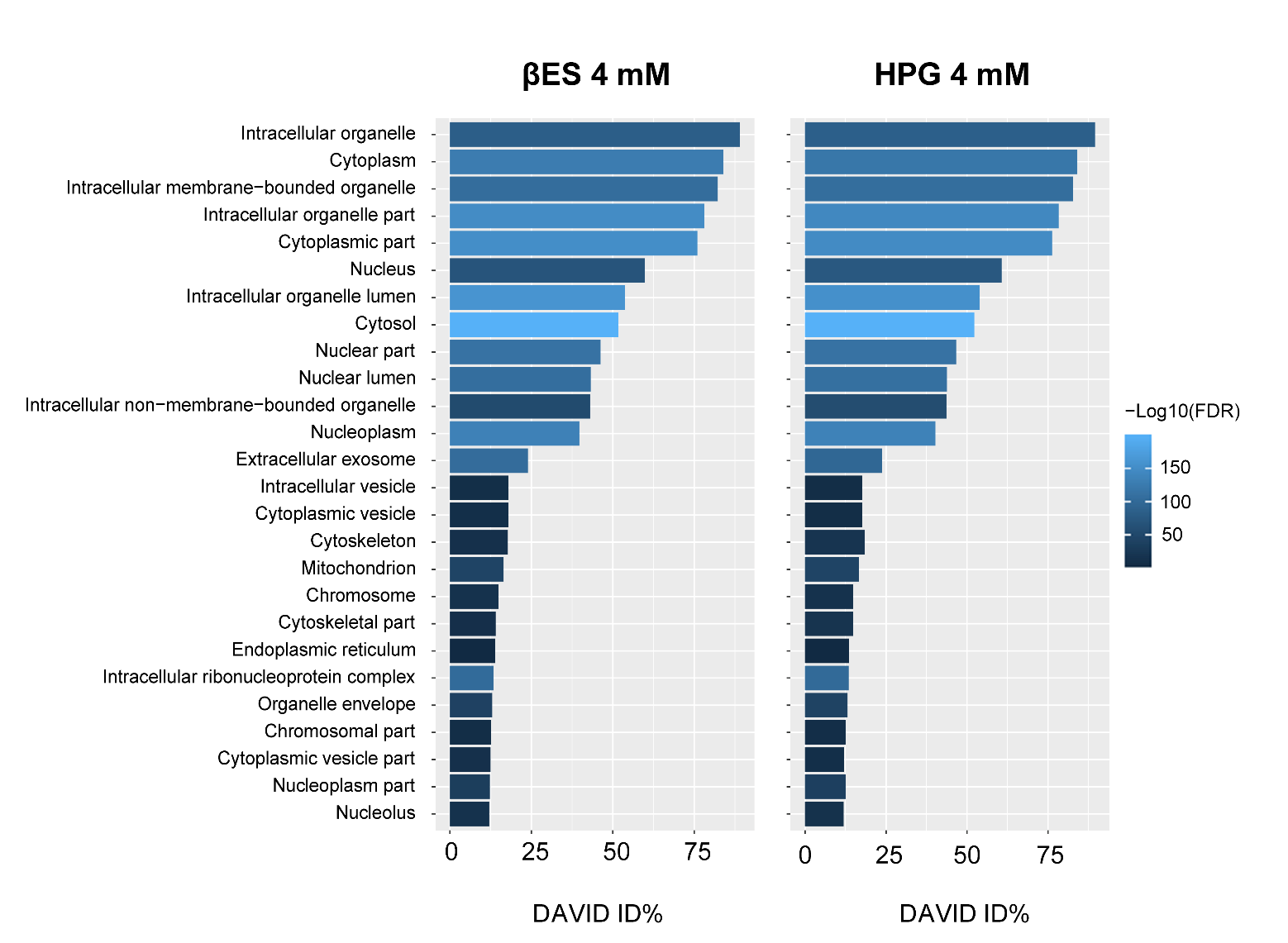


**Supplementary Figure 12: Bar chart showing the top enriched cellular compartments based on overrepresentation analysis**. Newly synthesized proteins identified by 4 mM βES labeling (THRONCAT, left panel) and by 4 mM HPG labeling (BONCAT, right panel) were used as input for overrepresentation analysis. DAVID ID%: percentage of input entries that are associated with a particular GO-term. FDR, False discovery rate.


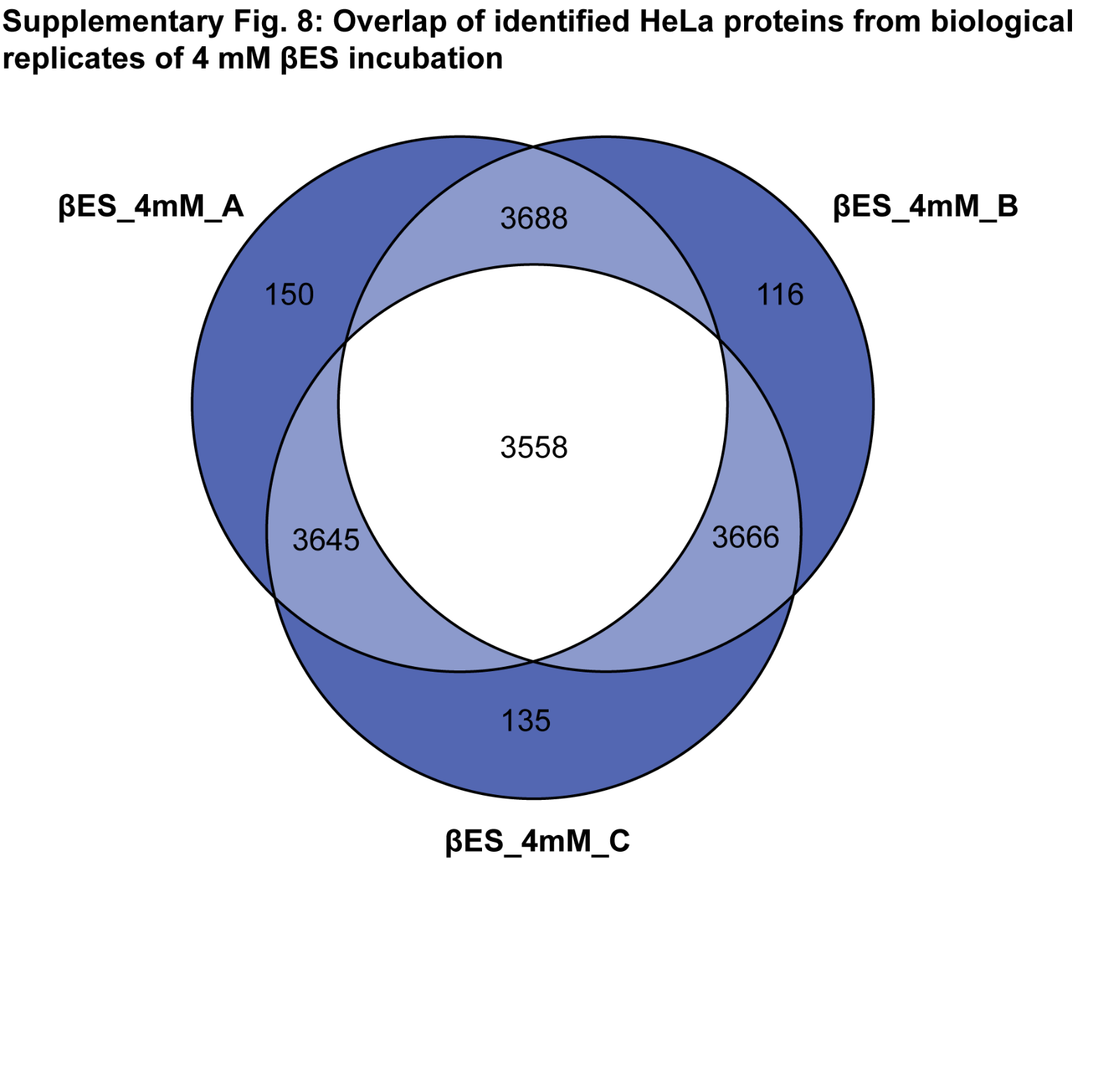


**Supplementary Figure 13: Overlap between HeLa proteins identified in three replicate THRONCAT experiments.** HeLa cells were incubated for 5 h with 4 mM βES in complete medium. NSPs were enriched from cell lysates, digested and peptides subjected to LC-MS/MS analysis. The number of identified proteins are indicated in the diagram. See Supplementary Data 2 for complete list of identified proteins.


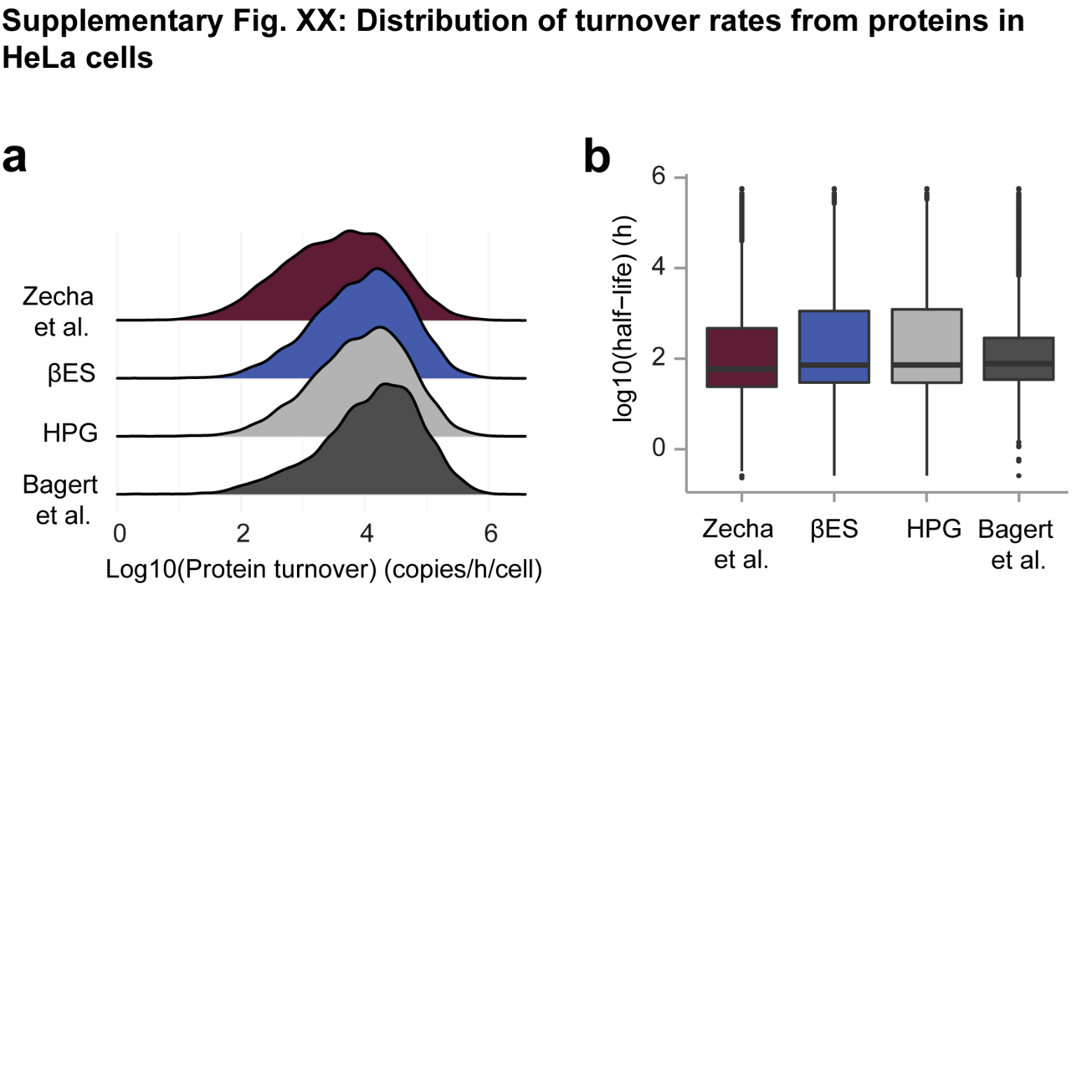


**Supplementary Figure 14: Distribution of turnover rates of newly synthesized proteins enriched by THRONCAT or BONCAT in HeLa cells**. **a**, Ridgeplots showing the protein turnover rates per hour, per cell for a dataset of 9688 HeLa proteins from Zecha *et al*.^1^ and newly synthesized proteins enriched by THRONCAT (4 mM βES) or BONCAT (4 mM HPG). A dataset from Bagert et al.^2^ was included for comparison, consisting of newly synthesized proteins enriched from HeLa cells incubated for 4 h with methionine analog azidohomoalanine (AHA). **b**, Boxplots showing the half-lives for the datasets as described for panel **a**. The boxplots are based on the following values for number of data points (*n*), minimal value (min), 1^st^ quartile, median, 3^rd^ quartile, maximal value (max). For Zecha *et al*.,^1^ *n* = 16675; min = −0.632, 1^st^ quartile = 1.38, median = 1.77, 3^rd^ quartile = 2.67, max = 5.76. For βES, *n* = 7711, min = −0.584, 1^st^ quartile = 1.47, median = 1.86, 3^rd^ quartile = 3.05, max = 5.76. For HPG, *n* = 7805, min = −0.584, 1^st^ quartile = 1.47, median = 1.86, 3^rd^ quartile = 3.09, max = 5.76. Bagert *et al.*,^2^ *n* = 2882. min = −0.584, 1^st^ quartile = 1.54, median = 1.89, 3^rd^ quartile = 2.46, max = 5.76. Whiskers represent 1.5x the interquartile range (IQR) for all datasets. βES, β-ethynyl serine; HPG, homopropargylglycine.


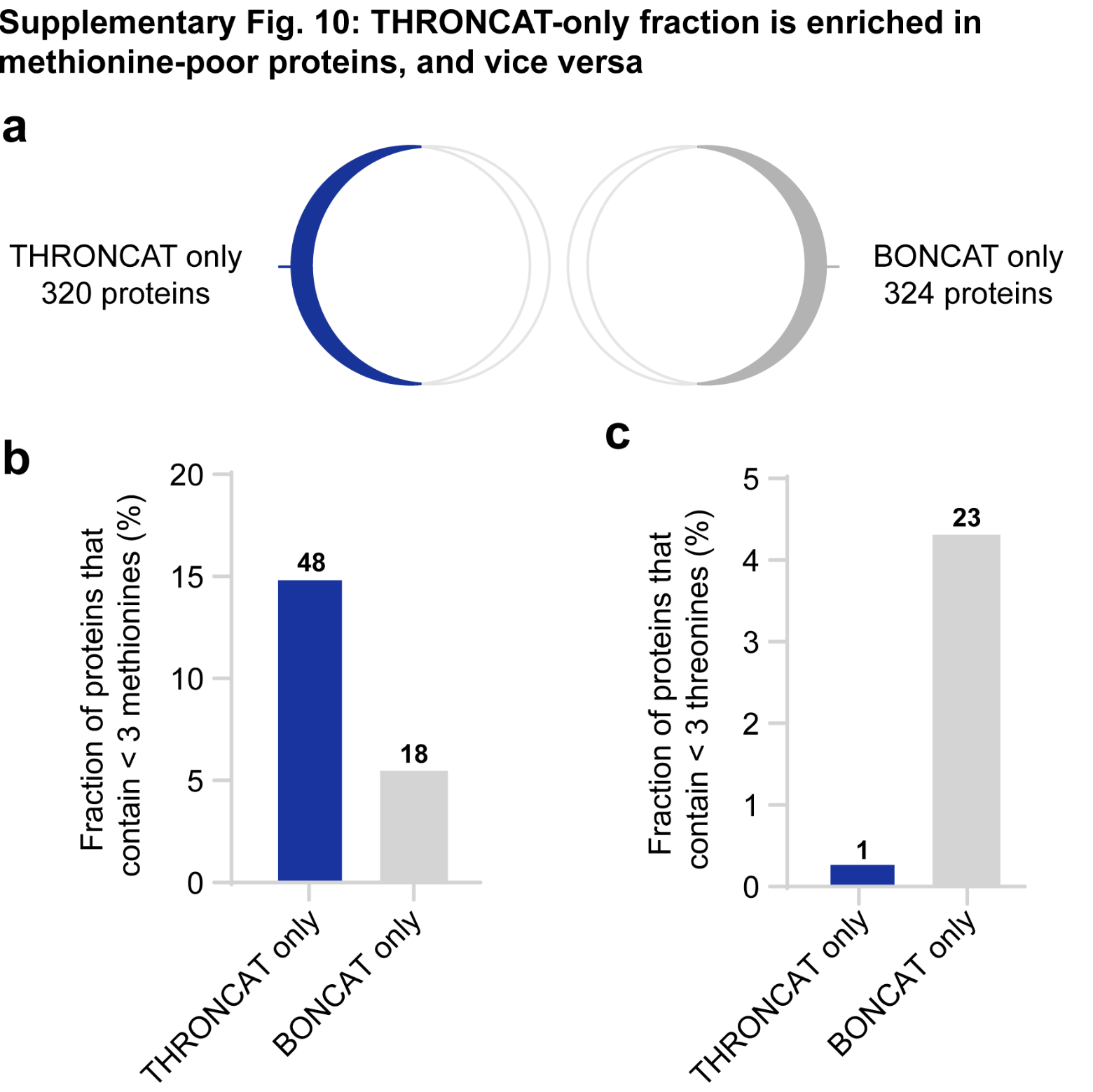


**Supplementary Figure 15: Analysis of protein composition of THRONCAT-only and BONCAT-only fractions. a,** Scheme showing THRONCAT-only and BONCAT-only fractions in Venn diagram from **Figure 4c**. **b,** Relative abundance of methionine-poor proteins in THRONCAT-only and BONCAT-only fractions. Methionine-poor proteins, containing less than 3 methionine residues, were enriched in the THRONCAT-only fraction. **c,** Relative abundance of threonine-poor proteins in THRONCAT-only and BONCAT-only fractions. Threonine-poor proteins, containing less than 3 threonine residues, were enriched in the BONCAT-only fraction. **b-c**, Protein composition was analyzed using a custom-made python script (Supplementary Software). Numbers above bars represent the absolute number of methionine- or threonine-poor proteins identified in each fraction.

**­
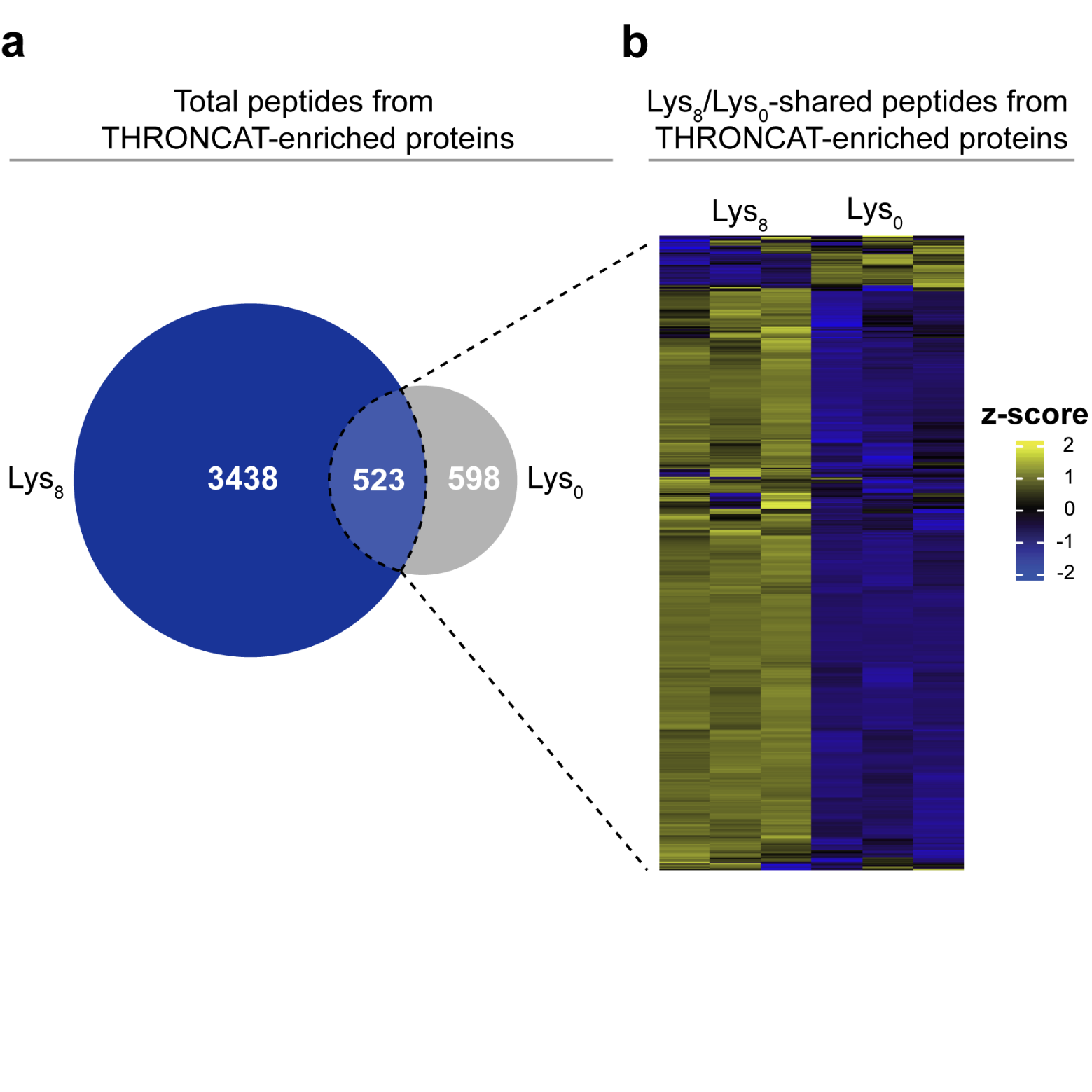
**

**Supplementary Figure 16: Lys_8_-labeled peptides are overrepresented in THRONCAT-enriched proteins from Ramos cells. a**, ­Venn diagram showing the number of Lys_8_- or Lys_0_-modified peptides in THRONCAT-enriched proteins from Ramos B cells. The number of peptides is indicated in white within the sections of the Venn diagram. Of the total number of peptides identified using THRONCAT, 86.9% contained a Lys_8_-modification. The Venn diagram shows peptides that were detected in 3 biological replicates. **b**, Heatmap showing the relative abundance of 523 peptides found with Lys_8_- and Lys_0_-modifications. In the majority of the shared peptides, Lys_8_-modified peptides are considerably more abundant than Lys_0_-modified peptides.

**
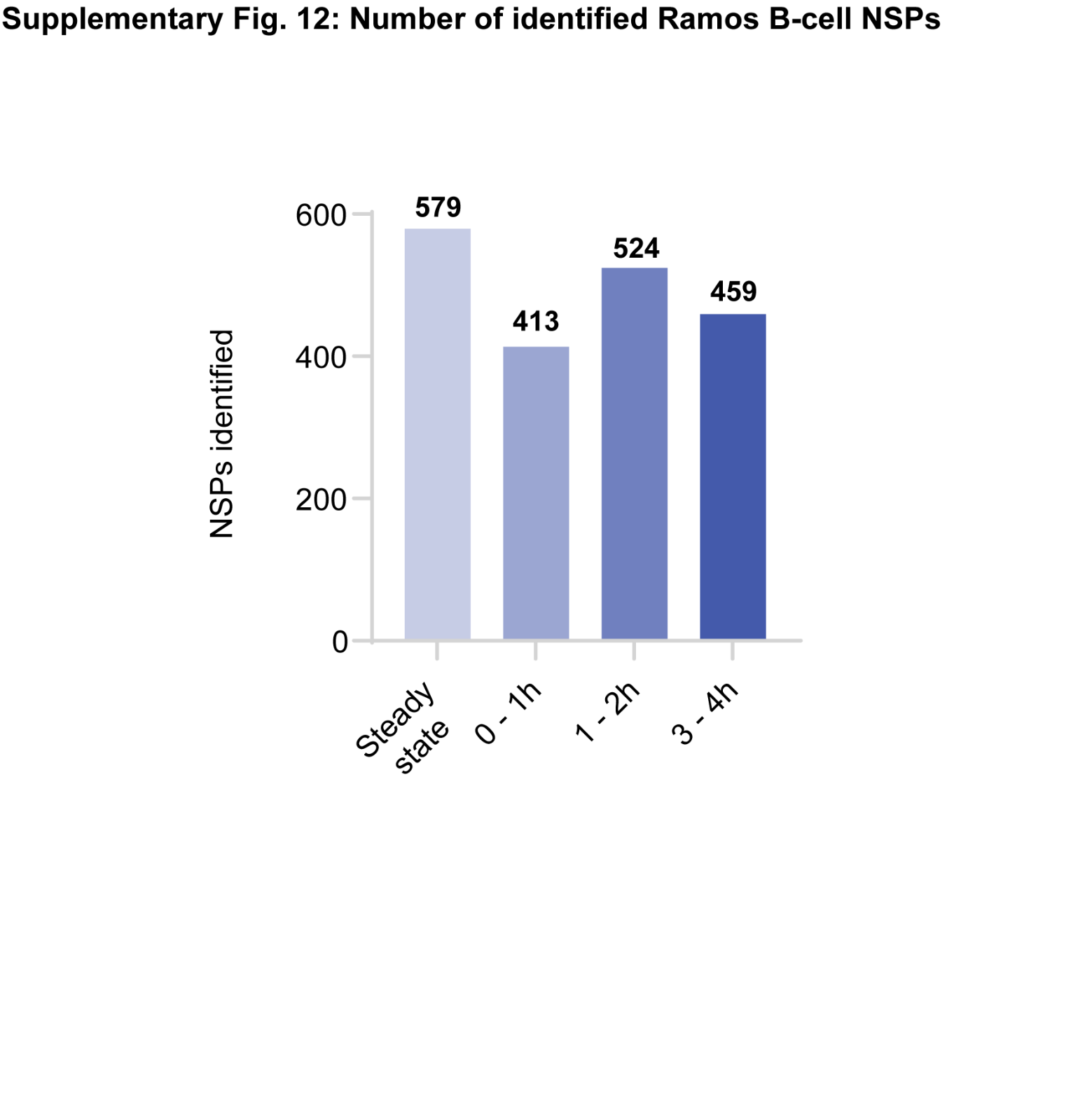
**

**Supplementary Figure 17: NSPs confidently identified in different time frames after Ramos cell activation.** Ramos B cells were pulse-labeled with 1 mM βES and d_8_-lysine for 1 h. NSPs were enriched, digested and subjected to LC-MS/MS analysis. Only peptides containing d_8_-lysine were used for protein identification and proteins were confidently identified as NSPs if they were present in all three biological replicates.


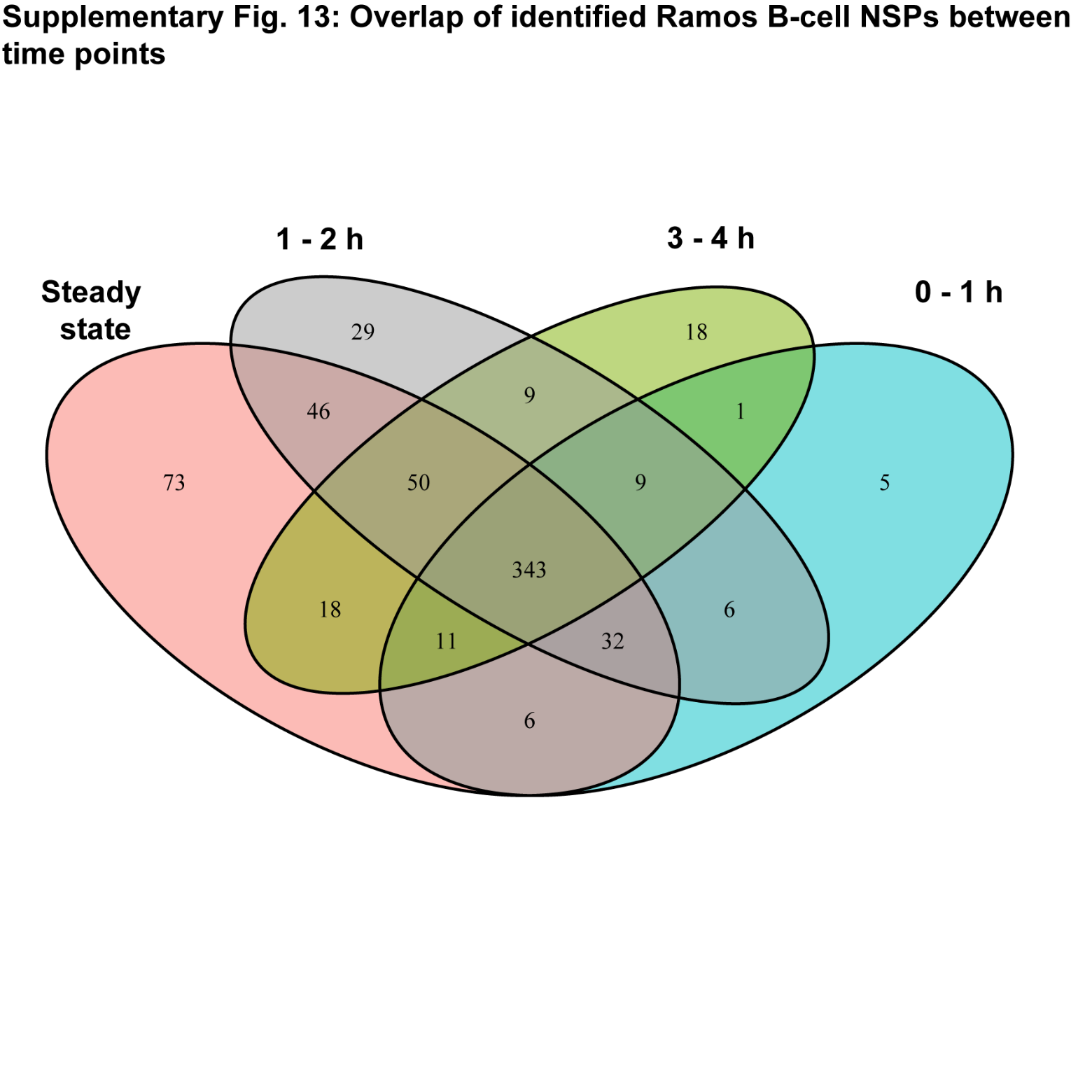


**Supplementary Figure 18: Activation of Ramos B cells induces significant proteomic changes.** Venn diagram showing the overlap in number of NSPs identified at steady state and at different time points using during the activation of Ramos B cells by THRONCAT.


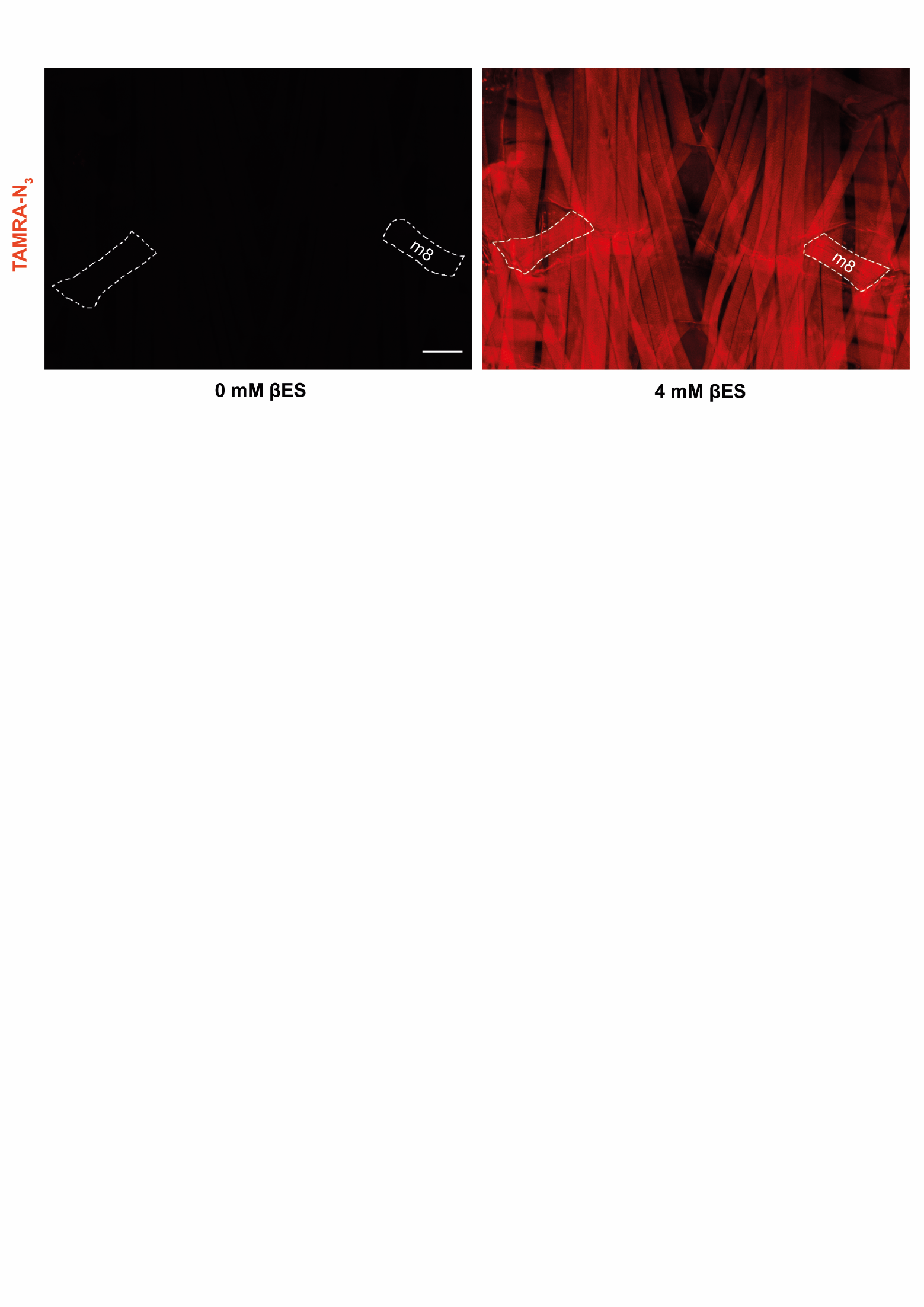


**Supplementary Figure 19:** **Representative images of in vivo THRONCAT in body wall muscles of Drosophila larvae.** Larvae were exposed to medium containing either 0 mM (control) or 4 mM βES, followed by conjugation to TAMRA-N3. Muscle 8 (m8) is delineated. Scale bar: 100 μm. βES, β-ethynyl serine. The experiments were repeated 6 (0 mM) and 3 (4 mM) times, representative images are showed here.


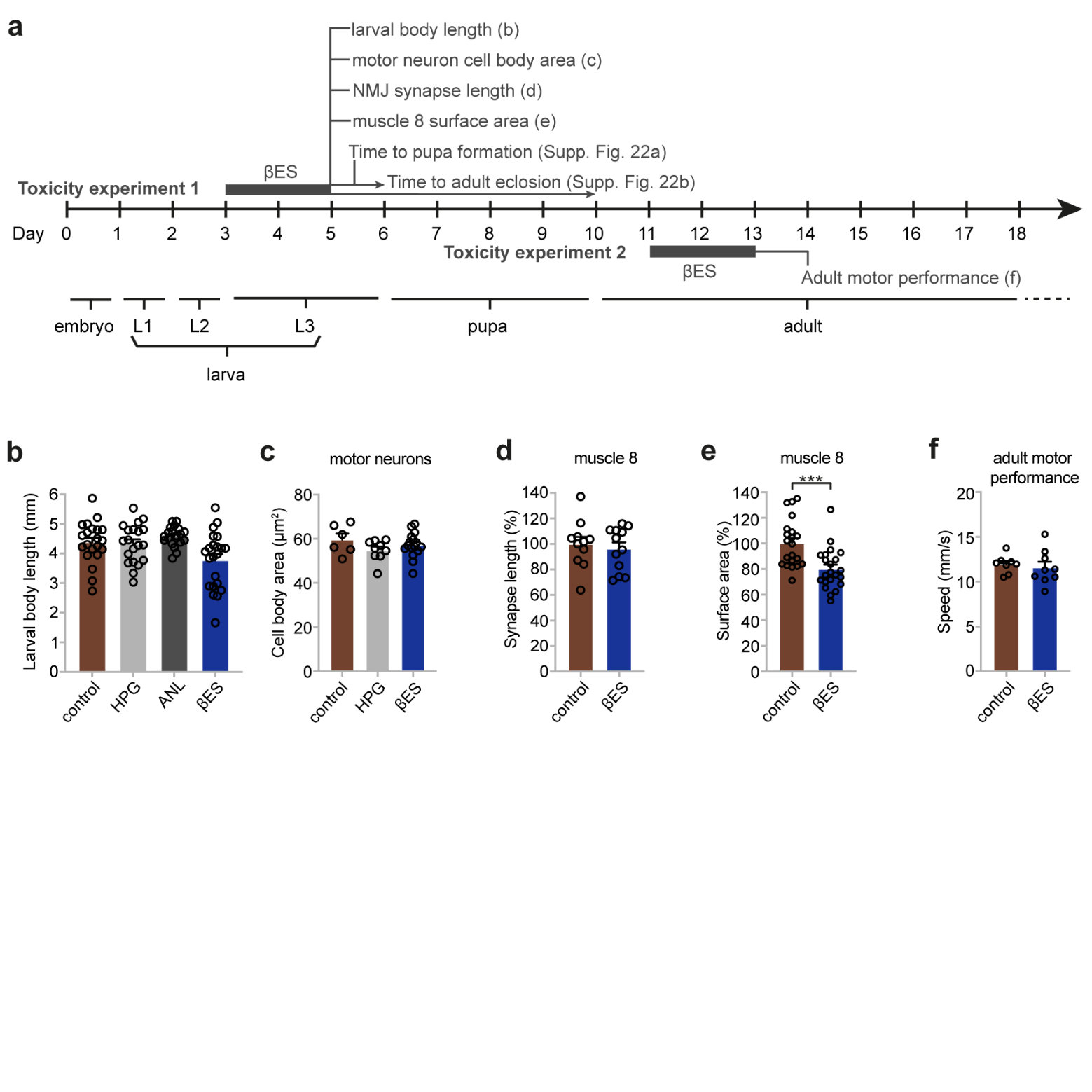


**Supplementary Figure 20:** **Evaluation of toxicity of βES administration in *Drosophila*. a**, Schematic representation of the experiments designed to evaluate toxicity of βES administration to larvae (experiment 1) and adult flies (experiment 2). The 48 h βES administration window is represented by filled rectangles. See Supplementary Discussion 1 for details. **b,** Body length of larvae exposed to control medium or medium containing 4 mM HPG, 4 mM ANL or 4 mM βES for 48 h. *n* = 21 (control), 22 (HPG), 20 (ANL) or 20 (βES) larvae per treatment; *P* = not significant by Brown-Forsythe and Welch ANOVA. **c**, Motor neuron cell body area in larvae exposed to control, HPG- and βES-containing medium for 48 h. *n* = 6 (control), 9 (HPG) or 16 (βES) larvae per treatment; *P* = not significant by ordinary one-way ANOVA. **d**, Neuromuscular synapse length (% of control) on muscle 8 of larvae exposed to control and βES-containing medium. *n* = 11 (control) or 12 (βES) larvae per treatment; *P* = not significant by unpaired two-tailed t-test. **e**, Surface area (% of control) of muscle 8 of larvae exposed to control and βES-containing medium. *n* = 21 (control) or 22 (βES) larvae per treatment; ****P* = 0.0005 by unpaired two-tailed t-test. **f**, Motor performance as evaluated by climbing speed in an automated negative geotaxis essay of adult flies exposed to control or βES-containing medium for 48 h. *n* = 8 (control) or 9 (βES) groups of 10 flies per treatment; *P* = not significant by unpaired two-tailed t-test. Error bars in **b-f** represent SEM. NMJ, neuromuscular junction; βES, β-ethynyl serine; HPG, homopropargylglycine; ANL, azidonorleucine.

­­­­
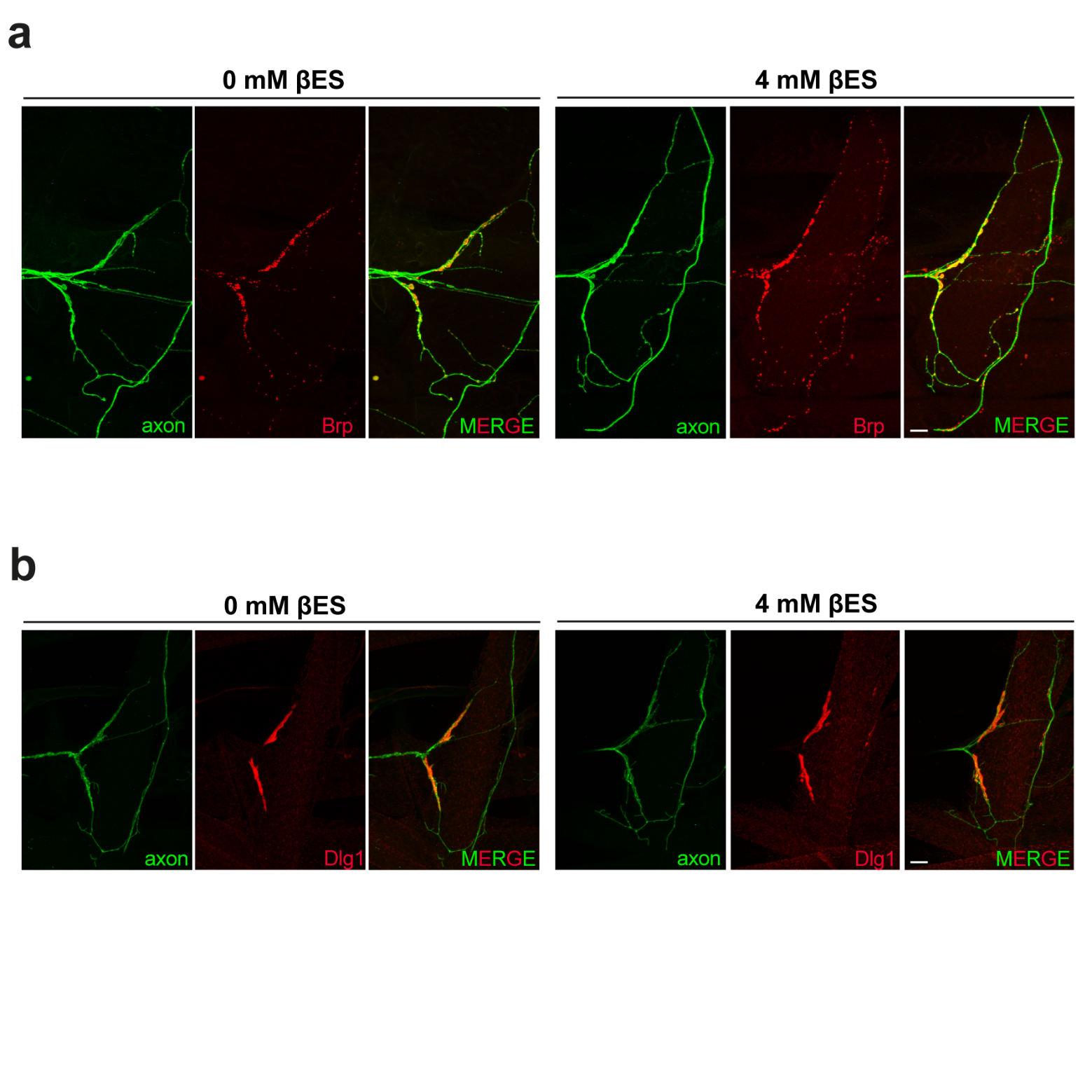


**Supplementary Figure 21: βES administration does not affect larval neuromuscular junction morphology. a**, Representative images of immunostaining for the presynaptic active zone marker Brp in larvae that selectively express membrane-tethered GFP in motor neurons (OK371-GAL4>UAS-mCD8::GFP). Treatment of larvae with 4 mM βES for 48 h did not affect the morphology of the presynaptic nerve terminal, including the distribution of active zones. **b**, Representative images of immunostaining for the postsynaptic marker Dlg1 in larvae that selectively express membrane-tethered GFP in motor neurons (OK371-GAL4>UAS-mCD8::GFP). Treatment of larvae with 4 mM βES for 48 h did neither affect the morphology of the presynaptic nerve terminal, nor the distribution of the postsynaptic marker Dlg1 compared to untreated larvae (control). βES, β-ethynyl serine. Scale bar: 10 μm. The experiments were repeated 4 (0 mM, Brp), 5 (4 mM, Brp), 11 (0 mM, Dlg1) and 12 (4 mM, Dlg1) times, representative images are showed here.


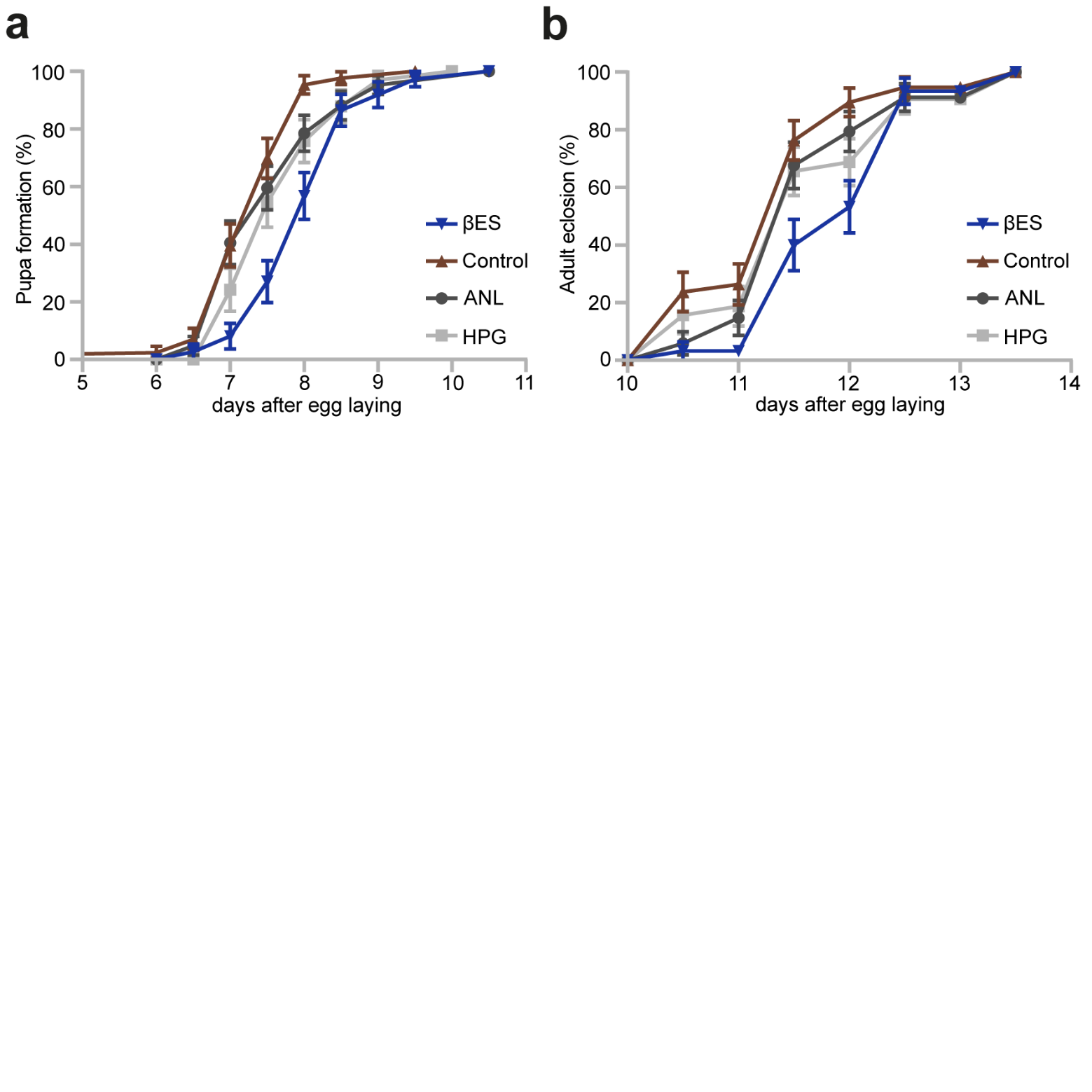


**Supplementary Figure 22: βES administration induces a slight delay in *Drosophila* development.** Percentage of pupae formed (**a**) and adult flies eclosed from the pupal case (**b**) in function of days after egg laying. Larvae exposed to control medium were compared to larvae exposed to medium containing 4 mM HPG, 4 mM ANL, or 4 mM βES for 48 h. **a**, *n* = 37 (βES), 43 (control), 42 (ANL) or 33 (HPG) larvae per treatment. **b**, *n* = 29 (βES), 37 (control), 33 (ANL) or 31 (HPG) larvae per treatment; Bonferroni corrected *P*-values as determined by Log-rank (Mantel-Cox) test: (**a**) *P* < 0.0005 for control versus βES, *P* = 0.136 for control versus HPG, *P* = 0.6025 control versus ANL, *P* = 0.333 HPG versus βES and *P* = 0.2055 ANL versus βES; (**b**) *P* = 0.006 for control versus βES, *P* = 0.608 for control versus HPG, *P* = 0.9245 control versus ANL, *P* > 0.9999 HPG versus βES and *P* = 0.33 ANL versus βES. Error bars represent SEM. βES, β-ethynyl serine; HPG, homopropargylglycine; ANL, azidonorleucine.

**
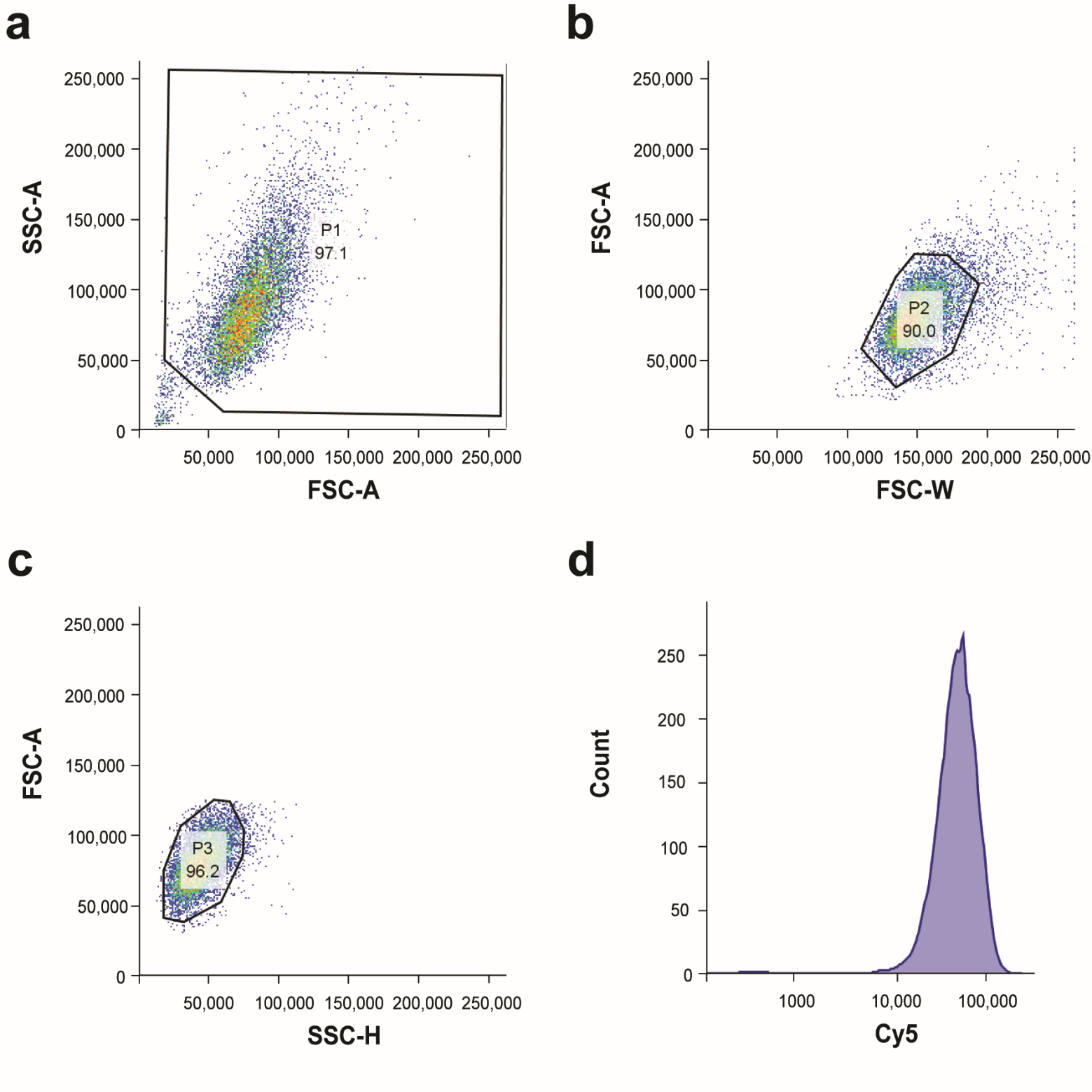
**

**Supplementary Figure 23: Flow cytometry plots exemplifying the gating strategy used throughout this work.** **a,** Dot plot showing side scatter area (SSC-A) vs. forward scatter area (FSC-A) of all acquired data points. A wide gate was applied, yielding child population P1. **b**, Dot plot showing FSC-A vs. forward scatter width (FSC-W) of population P1. A tight gate was applied, yielding child population P2. **c,** Dot plot showing FSC-A vs. side scatter height (SSC-H) of population P2. A tight gate was applied, yielding child population P3. **d,** Histogram showing the distribution of Cy5 fluorescence intensity in population P3. Mean fluorescence intensity was determined by averaging the fluorescence intensity of population P3.

| Treatment | Time points | p-value | p-value summary |
| --- | --- | --- | --- |
| βES | 2h versus 4h | 0.012 | * |
|  | 2h versus 8h | 0.0012 | *** |
|  | 2h versus 16h | <0.0001 | *** |
|  | 2h versus 48h | <0.0001 | *** |
|  | 4h versus 8h | 0.37 | ns |
|  | 4h versus 16h | <0.0001 | *** |
|  | 4h versus 48h | <0.0001 | *** |
|  | 8h versus 16h | 0.0001 | *** |
|  | 8h versus 48h | <0.0001 | *** |
|  | 16h versus 48h | <0.0001 | *** |
| HPG | 2h versus 4h | >0.99 | ns |
|  | 2h versus 8h | 0.24 | ns |
|  | 2h versus 16h | 0.0004 | *** |
|  | 2h versus 48h | <0.0001 | *** |
|  | 4h versus 8h | >0.99 | ns |
|  | 4h versus 16h | 0.020 | * |
|  | 4h versus 48h | 0.0004 | *** |
|  | 8h versus 16h | 0.52 | ns |
|  | 8h versus 48h | 0.033 | * |
|  | 16h versus 48h | >0.99 | ns |

**Supplementary Table 1:** Statistics to show that THRONCAT signal intensity in *Drosophila* motor neurons increases with increasing labeling times. Brown-Forsythe ANOVA with Dunnett’s T3 multiple comparisons test was performed for βES time points. Kruskal-Wallis test with Dunn’s multiple comparisons test was performed for HPG time points. ns, not significant.

|  | **Threonine-free medium** | | **Methionine-free medium** | |
| --- | --- | --- | --- | --- |
|  | Concentration  (mg/L) | Concentration  (mM) | Concentration (mg/L) | Concentration  (mM) |
| **Amino acids** | | | | |
| Glycine | 30 | 0,4 | 30 | 0,4 |
| L-Arginine HCl | 84 | 0,398104 | 84 | 0,398104 |
| L-Cystine 2 HCl | 63 | 0,201278 | 63 | 0,201278 |
| L-Glutamine | 584 | 4 | 584 | 4 |
| L-Histidine hydrochloride-H_2_O | 42 | 0,2 | 42 | 0,2 |
| L-Isoleucine | 105 | 0,801527 | 105 | 0,801527 |
| L-Leucine | 105 | 0,801527 | 105 | 0,801527 |
| L-Lysine HCl | 146 | 0,797814 | 146 | 0,797814 |
| L-Methionine | 30 | 0,201342 | - | - |
| L-Phenylalanine | 66 | 0,4 | 66 | 0,4 |
| L-Serine | 42 | 0,4 | 42 | 0,4 |
| L-Threonine | - | - | 95 | 0,798319 |
| L-Tryptophan | 16 | 0,078431 | 16 | 0,078431 |
| L-Tyrosine disodium salt dihydrate | 104 | 0,398467 | 104 | 0,398467 |
| L-Valine | 94 | 0,803419 | 94 | 0,803419 |
| **MEM vitamine mix. 1x, diluted from 100x (Fisher Scientific, Cat. No.: 11120037)** | | | | |
| Choline chloride | 1 | 0,007143 | 1 | 0,007143 |
| D-Calcium pantothenate | 1 | 0,002096 | 1 | 0,002096 |
| Folic Acid | 1 | 0,002268 | 1 | 0,002268 |
| Nicotinamide | 1 | 0,008197 | 1 | 0,008197 |
| Pyridoxal HCl | 1 | 0,004854 | 1 | 0,004854 |
| Riboflavin | 0,1 | 0,000266 | 0,1 | 0,000266 |
| Thiamine HCl | 1 | 0,002967 | 1 | 0,002967 |
| i-Inositol | 2 | 0,011111 | 2 | 0,011111 |
| **Earl's balanced salt solution (EBSS, Fisher Scientific, Cat. No.: 11540616)** | | | | |
| Calcium Chloride (CaCl_2_) (anhydr.) | 200 | 1,801802 | 200 | 1,801802 |
| Magnesium Sulfate (MgSO_4_-7H_2_O) | 200 | 0,813008 | 200 | 0,813008 |
| Potassium Chloride (KCl) | 400 | 5,333333 | 400 | 5,333333 |
| Sodium Bicarbonate (NaHCO3) | 2200 | 26,19048 | 2200 | 26,19048 |
| Sodium Chloride (NaCl) | 6808,5 | 117,3879 | 6808,5 | 117,3879 |
| Sodium Phosphate monobasic (NaH_2_PO_4_-H_2_O) | 140 | 1,014493 | 140 | 1,014493 |
| D-Glucose (Dextrose) | 4500 | 25 | 4500 | 25 |
| Phenol Red | 10 | 0,025126 | 10 | 0,025126 |

**Supplementary Table 2. Formulations of threonine-free medium and methionine-free medium based on Dulbecco’s modified Eagle’s medium (DMEM)**. Earl’s balanced salt solution was used as base for the custom media. Amino acids, D-glucose and MEM vitamin mix (100x) were added to the final concentrations outlined in the table. Threonine was omitted in threonine-free medium (green) and methionine was omitted in methionine-free medium (blue).

# **Supplementary Discussion 1: Evaluation of toxicity from βES administration in *Drosophila***

We performed two experiments to evaluate whether βES incorporation would induce toxicity in *Drosophila* (Supplementary Figure 20a). In the first experiment, we exposed wild type late second instar/early third instar larvae to medium containing 4 mM βES, 4 mM HPG, 4 mM ANL, or control medium for 48 h. At the end of this incubation period, we determined the larval body length (Supplementary Figure 20b), the surface area of motor neuron cell bodies (Supplementary Figure 20c), the synapse length of the neuromuscular junction (NMJ) on larval muscle 8 (Supplementary Figure 20d), the surface area of muscle 8 (Supplementary Figure 20e), and NMJ morphology (Supplementary Figure 21). For a separate cohort of larvae, we determined the effect of βES, HPG, or ANL administration on the time to pupa formation and adult eclosion (Supplementary Figure 22).

None of the non-canonical amino acids induced lethality of larvae during the 48 h incubation period. Whereas HPG and ANL administration (to larvae that do not express MetRS^L262G^) did not affect larval body length, βES administration tended to reduce body length although this difference did not reach statistical significance (Supplementary Figure 20b, *P* = 0.07 for control versus βES by Brown-Forsythe and Welch ANOVA). Neither βES nor HPG administration affected motor neuron cell body area (Supplementary Figure 20c), and βES exposure also did not affect NMJ synapse length (Supplementary Figure 20d), while the surface area of larval muscle 8 was reduced by 20% (Supplementary Figure 20e). To evaluate NMJ morphology, we performed immunostaining for the presynaptic active zone marker Brp and the postsynaptic marker Dlg1 in larvae that selectively express membrane-tethered GFP in motor neurons. As compared to control medium, βES treatment affected neither the morphology of the presynaptic nerve terminal including the distribution of active zones (Supplementary Figure 21a), nor the distribution of the postsynaptic marker Dlg1 (Supplementary Figure 21b). Finally, we evaluated whether treating larvae with 4 mM βES for 48 h would induce a developmental delay. While HPG and ANL treatment did not significantly increase the time to pupa formation, βES treatment slightly increased the median time to pupa formation by 12 h, and this difference reached statistical significance (Supplementary Figure 22a). βES treatment also delayed the median time to adult eclosion by 12 h, whereas HPG and ANL treatment did not significantly alter the time to adult eclosion (Supplementary Figure 22b). Thus, the slight developmental delay incurred during larval life upon βES exposure did not increase further during metamorphosis.

In the second experiment (Supplementary Figure 20a), we exposed 1-day-old adult flies to 4 mM βES for 48 h, followed by incubation for 24 h on regular food and evaluation of motor performance. The climbing speed of flies in an automated negative geotaxis climbing assay^3^ was not affected by βES treatment as compared to flies exposed to control food (Supplementary Figure 20f). Taken together, these results indicate that βES administration to larvae during a 48 h time frame reduces larval growth and induces a slight developmental delay, while βES administration to adult flies did not induce significant toxicity.

# **Supplementary Note 1: Detailed description of organic syntheses**

## **General synthetic methods**

^1^H and ^13^C NMR spectra were recorded on a Bruker 400 MHz or 500 MHz spectrometer. Chemical shifts are reported in parts per million (ppm), using tetramethylsilane (TMS) or residual solvents as the internal standard. NMR data is presented as follows: chemical shift, multiplicity, coupling constant in hertz (Hz), integration. All NMR signals were assigned on the basis of ^1^H NMR, ^13^C APT NMR, COSY, HSQC and HMBC experiments. Mass spectra were recorded on a JEOL AccuTOF CS JMST100CS mass spectrometer. Optical rotation was measured on an Anton Paar MCP 100 polarimeter. Automatic flash column chromatography was performed using a Biotage Isolera Spektra One. TLC analysis was conducted on Silicagel F254 (Merck KGaA) with detection by UV-absorption (254nm) or staining with ninhydrin or KMnO_4_ solutions. DCM and THF were freshly distilled. All inert reactions were carried out under nitrogen atmosphere using flame-dried flasks.

**(9H-fluoren-9-yl)methyl chlorocarbamate (5)**

**5** was synthesized according to a previously described procedure.^4^ Briefly, 9-Fluorenylmethyl Carbamate (2.28 g, 9.55 mmol) was dissolved with heating in MeOH (170 mL). When the reaction mixture had cooled to 35 °C, trichloroisocyanuric acid (737 mg, 3.17 mmol) was added in one portion. The reaction mixture was stirred at room temperature for 18 hours, when another portion of trichloroisocyanuric acid (207 mg, 0.891 mmol) was added. After stirring for another 4.5 hours at room temperature, the reaction mixture was concentrated *in vacuo*. The resulting solid was suspended in hot toluene and then filtered while hot. Upon cooling, crystals appeared in the filtrate. The filtrate was heated and then allowed to slowly cool to room temperature, followed by cooling to 4 °C overnight. The resulting crystals were filtered and dried under high vacuum to yield **5** (2.52 g, 97%) as a white fluffy solid. **TLC** (EtOAc/*n*-heptane, 1/1, v/v): R_f_ = 0.68; **^1^H NMR** (400 MHz, CDCl_3_) δ 7.77 (dt, J = 7.6, 1.0 Hz, 2H), 7.61 (dq, J = 7.5, 1.0 Hz, 2H), 7.44 – 7.39 (m, 2H), 7.33 (td, J = 7.5, 1.2 Hz, 2H), 5.56 (s, 1H), 4.51 (d, J = 7.1 Hz, 2H), 4.26 (t, J = 6.9 Hz, 1H); **^13^C NMR** (101 MHz, CDCl_3_) δ 156.70, 143.22, 141.35, 127.97, 127.21, 125.05, 120.11, 70.44, 46.85; **HRMS** (m/z): [M + Na]^+^ calculated for C_15_H_12_ClNO_2_, 296.0454; found, 296.0455.

**methyl (E)-5-(trimethylsilyl)pent-2-en-4-ynoate (2)**

3-(Trimethylsilyl)-2-propynal (1.20 mL, 8.15 mmol) was dissolved in dry THF (35 mL) under inert atmosphere and cooled to 0 °C. Methyl (triphenylphosphoranylidene)acetate (3.28 g, 9.82 mmol) was added in one portion and the resulting mixture was stirred at 0 °C for 1.5 hours. The reaction mixture was concentrated *in vacuo* and silica column chromatography (0 – 10% Et_2_O in *n*-pentane) afforded the title compound **2** (1.41 g, 95%) as a clear liquid. **TLC** (EtOAc/*n*-heptane, 1/4, v/v): R_f_ = 0.67; **^1^H NMR** (400 MHz, CDCl_3_) δ 6.73 (d, J = 15.9 Hz, 1H), 6.24 (d, J = 16.0 Hz, 1H), 3.74 (s, 3H), 0.20 (s, 9H); **^13^C NMR** (101 MHz, CDCl_3_) δ 167.19, 133.40, 128.58, 105.49, 101.65, 52.71, -0.003; **HRMS** (m/z): [M + H]^+^ calculated for C_9_H_14_O_2_Si, 183.0841; found, 183.0835.

**methyl (2S,3R)-2-((((9H-fluoren-9-yl)methoxy)carbonyl)amino)-3-hydroxy-5-(trimethylsilyl)pent-4-ynoate (3)**

## To a stirring suspension of **5** (3.21 g, 11.7 mmol) in *n*-PrOH (12 mL) cooled to 0 °C was added NaOH (359 mg, 8.98 mmol) in H_2_O (15 mL). To this mixture was added a solution of (DHQD)_2_AQN (601 mg, 0.704 mmol) in *n*-PrOH (18 mL), followed by a solution of **2** (1.09 g, 5.98 mmol) in *n*-PrOH (10 mL). At this point, the reaction mixture was a homogenous, bright yellow solution. A solution of K_2_OsO_4_ · 2 H_2_O (176 mg, 0.479 mmol) in H_2_O (5 mL, a few drops of the NaOH solution were added to the solution of K_2_OsO_4_ · 2 H_2_O) was added to the reaction mixture, upon which the color of the reaction mixture turned to deep green. The reaction mixture was stirred for 4 hours at 0 °C when the color of the reaction mixture had turned from deep green to yellow again and TLC indicated completion. The reaction mixture was diluted with EtOAc (30 mL) and then washed with sat. aq. Na_2_S_2_O_3_ (50 mL), sat. aq. NaHCO_3_ (50 mL) and brine (50 mL). The organic layer was dried over MgSO_4_, filtered and concentrated *in vacuo*. Purification over silica column chromatography (10 – 25% EtOAc in *n­*-heptane), followed by recrystallization from *n*-heptane yielded title compound **3** (803 mg, 31%, 86% *ee*) as a white crystalline solid. **TLC** (EtOAc/n-heptane, 1/1, v/v): 0.55; **Specific rotation** [α]­_D_^20^ +21.9 (*c* 1.9, CH_2_Cl_2_); **^1^H NMR** (400 MHz, CDCl_3_) δ 7.77 (m, J = 7.6, 1.0 Hz, 2H), 7.62 (m, J = 7.6 Hz, 2H), 7.41 (m, J = 7.5, 1.0 Hz, 2H), 7.36 – 7.29 (m, 2H), 5.63 (d, J = 9.0 Hz, 1H), 4.81 (dd, J = 6.9, 3.4 Hz, 1H), 4.64 (dd, J = 9.1, 3.4 Hz, 1H), 4.41 (d, J = 7.3 Hz, 2H), 4.26 (t, J = 7.1 Hz, 1H), 3.81 (s, 3H), 2.74 (d, J = 6.8 Hz, 1H), 0.15 (s, 9H); **^13^C NMR** (101 MHz, CDCl_3_) δ 170.22, 144.07, 141.64, 128.10, 127.44, 125.46, 120.35, 67.82, 64.02, 58.82, 53.22, 47.43, 0.00; **HRMS** (m/z): [M + Na]^+^ calculated for C_24_H_27_NO_5_Si, 460.1556; found, 460.1554.

**methyl (2S,3R)-2-((((9H-fluoren-9-yl)methoxy)carbonyl)amino)-3-hydroxypent-4-ynoate (4)**

To a stirring solution of **3** (806 mg, 1.84 mmol) in DCM (15 mL) cooled to 0 °C was added TBAF (1M solution in THF; 3.68 mL, 3.68 mmol) dropwise. The reaction mixture was stirred for 10 minutes at 0 °C, when TLC indicated full conversion of **3**. The reaction mixture was diluted with EtOAc (10 mL) and washed with sat. aq. NH_4_Cl (2 x 25 mL) and brine (25 mL), dried over MgSO_4_, filtered and concentrated *in vacuo*. Purification over silica column chromatography (10 – 30 % EtOAc in *n*-heptane yielded title compound **4** (549 mg, 82 %) as a white crystalline solid. **TLC** (EtOAc/n-heptane, 1/1, v/v): R_f_ = 0.35; **Specific rotation** [α]­_D_^20^ +4.5 (*c* 2.2, CH_2_Cl_2_) **^1^H NMR** (400 MHz, CDCl_3_) δ 7.77 (dt, J = 7.6, 0.9 Hz, 2H), 7.62 (d, J = 7.5 Hz, 2H), 7.44 – 7.37 (m, 2H), 7.36 – 7.28 (m, 2H), 5.70 (d, J = 9.2 Hz, 1H), 4.86 (s, 1H), 4.66 (dd, J = 9.5, 3.1 Hz, 1H), 4.50 – 4.32 (m, 2H), 4.26 (t, J = 7.1 Hz, 1H), 3.81 (s, 3H), 3.04 (s, 1H), 2.49 (d, J = 2.2 Hz, 1H); **^13^C NMR** (101 MHz, CDCl_3_) δ 156.24, 143.70, 141.31, 127.76, 127.09, 125.12, 120.02, 80.52, 75.06, 63.05, 58.31, 53.01, 47.09, 30.95, 22.71, 14.13; **HRMS** (m/z): [M + Na]^+^ calculated for C_21_H_19_NO_5_, 388.1161; found, 388.1156.

**(2S,3R)-2-amino-3-hydroxypent-4-ynoic acid, β­-ethynylserine (1)**

To a stirring solution of **3** (526 mg, 1.44 mmol) in MeCN (10 mL) cooled to 0 °C was added a solution of LiOH (172 mg, 7.20 mmol) in H_2_O (10 mL) dropwise. The reaction mixture was stirred at 0 °C for 6 hours, when TLC indicated complete deprotection of the methyl ester and Fmoc-group. The mixture was filtered through a C18-funtionalized silica plug (Screening Devices). Dowex 50wx8 (Sigma, 10 mL) was washed with MeOH (50 mL) and H_2_O (50 mL), added to the reaction mixture and stirred at room temperature overnight. The mixture was filtered and the Dowex beads washed with 20 mL of a 3 M aq. NH_4_OH solution. The combined filtrate was concentrated *in vacuo* to yield title compound **1** (223 mg, 93%, >98% *de,* 86% *ee*) as a pale yellow, sticky solid. **Specific rotation** [α]­_D_^20^ – 52.9 (*c* 1.2, H_2_O); **^1^H NMR** (500 MHz, D_2_O) δ 5.12 (ddd, J = 3.0, 2.2, 0.8 Hz, 1H), 4.29 (dd, J = 3.1, 0.8 Hz, 1H), 3.12 (dd, J = 2.4, 1.0 Hz, 1H). **^13^C NMR** (126 MHz, D_2_O) δ 168.84, 78.67, 77.55, 59.47, 57.58. **HRMS** (m/z): [M + Na]^+^ calculated for C_5_H_7_NO_3_, 152.0324; found, 152.0346.

### **Mosher’s ester analysis for determination of absolute configuration** of **3**.^5^

To a stirred solution of **3** (9.1 mg, 20.8 μmol) in dry DCM (1 mL) was added, in this order, a catalytic amount of 4-dimethylaminopyridine (0.51 mg, 4.2 μmol), dry triethylamine (8.7 μL, 62 μmol) and *S*-(+)-MTPA-Cl (15.7 mg, 62 μmol). The reaction **mixture** was stirred at room temperature overnight, when TLC indicated full conversion of **3**. The reaction mixture was quenched by addition of sat. aq. NH_4_Cl (5 mL) and diluted with DCM (5 mL). The organic layer was washed with brine (5 mL), dried over MgSO_4_, filtered and dried *in vacuo*. Purification over silica column chromatography (0 – 20 % EtOAc in *n*-heptane) yielded the *R*-MTPA ester of **3** (7.4 mg, 55%) as a clear oil. **^1^H NMR** (500 MHz, CDCl_3_) δ 7.77 (ddt, J = 7.6, 3.2, 0.9 Hz, 2H), 7.59 (ddd, J = 11.8, 7.6, 3.1 Hz, 2H), 7.53 – 7.47 (m, 2H), 7.47 – 7.36 (m, 5H), 7.34 – 7.28 (m, 2H), 5.98 (d, J = 3.1 Hz, 1H), 5.50 (d, J = 9.7 Hz, 1H), 4.91 (dd, J = 9.7, 3.1 Hz, 1H), 4.37 (d, J = 7.5 Hz, 2H), 4.26 (dt, J = 14.7, 7.3 Hz, 1H), 3.74 (s, 3H), 3.54 – 3.50 (m, 3H), 0.10 (s, 9H). In an entirely analogous fashion, the *S*-MTPA ester of **3** was prepared from *R*-(-)-MPTA-Cl: **^1^H NMR** (500 MHz, CDCl_3_) δ 7.77 (dt, J = 7.6, 1.0 Hz, 2H), 7.62 – 7.55 (m, 2H), 7.55 – 7.48 (m, 2H), 7.48 – 7.37 (m, 5H), 7.30 (tdd, J = 7.5, 2.0, 1.2 Hz, 2H), 6.03 (d, J = 3.1 Hz, 1H), 5.45 (d, J = 9.7 Hz, 1H), 4.91 (dd, J = 9.7, 3.1 Hz, 1H), 4.39 – 4.34 (m, 2H), 4.26 (t, J = 7.5 Hz, 1H), 3.67 (s, 3H), 3.56 (s, 3H), 0.13 (s, 9H).

### **Supplementary Figure 24: NMR spectra of compound 4**

400 MHz, CDCl_3_


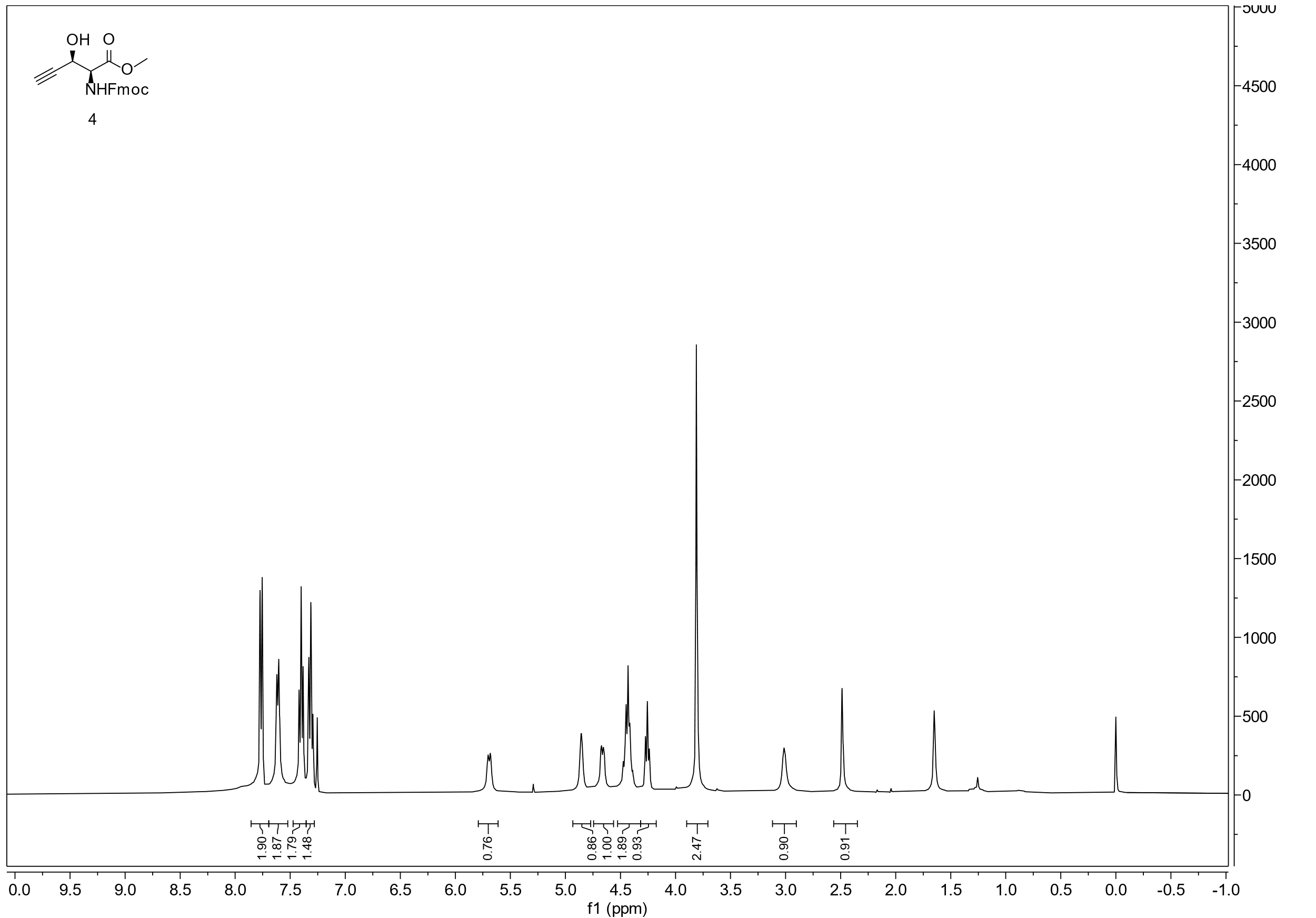


101 MHz, CDCl_3_


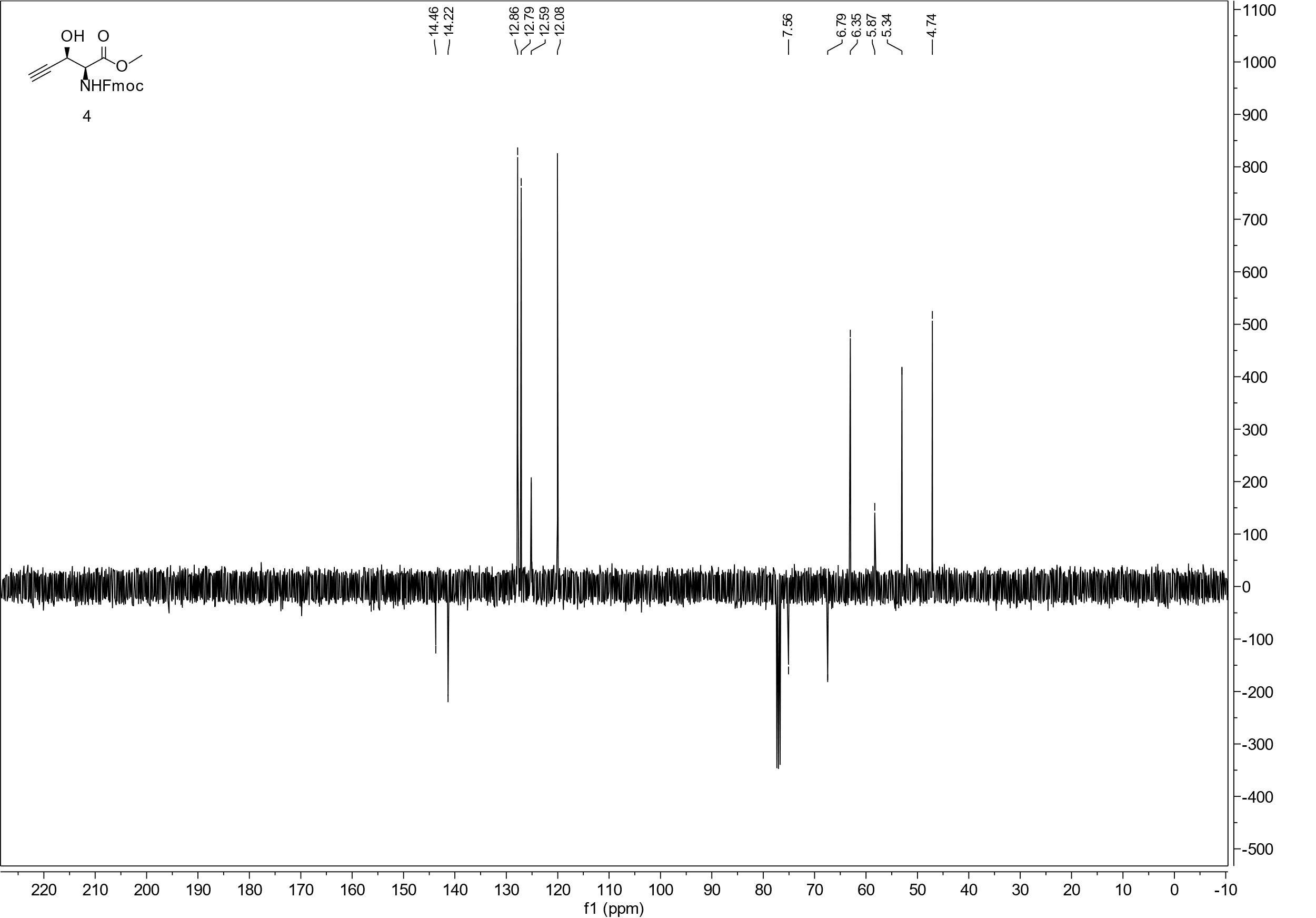


### **Supplementary Figure 25: NMR spectra of compound 5**

400 MHz, CDCl_3_


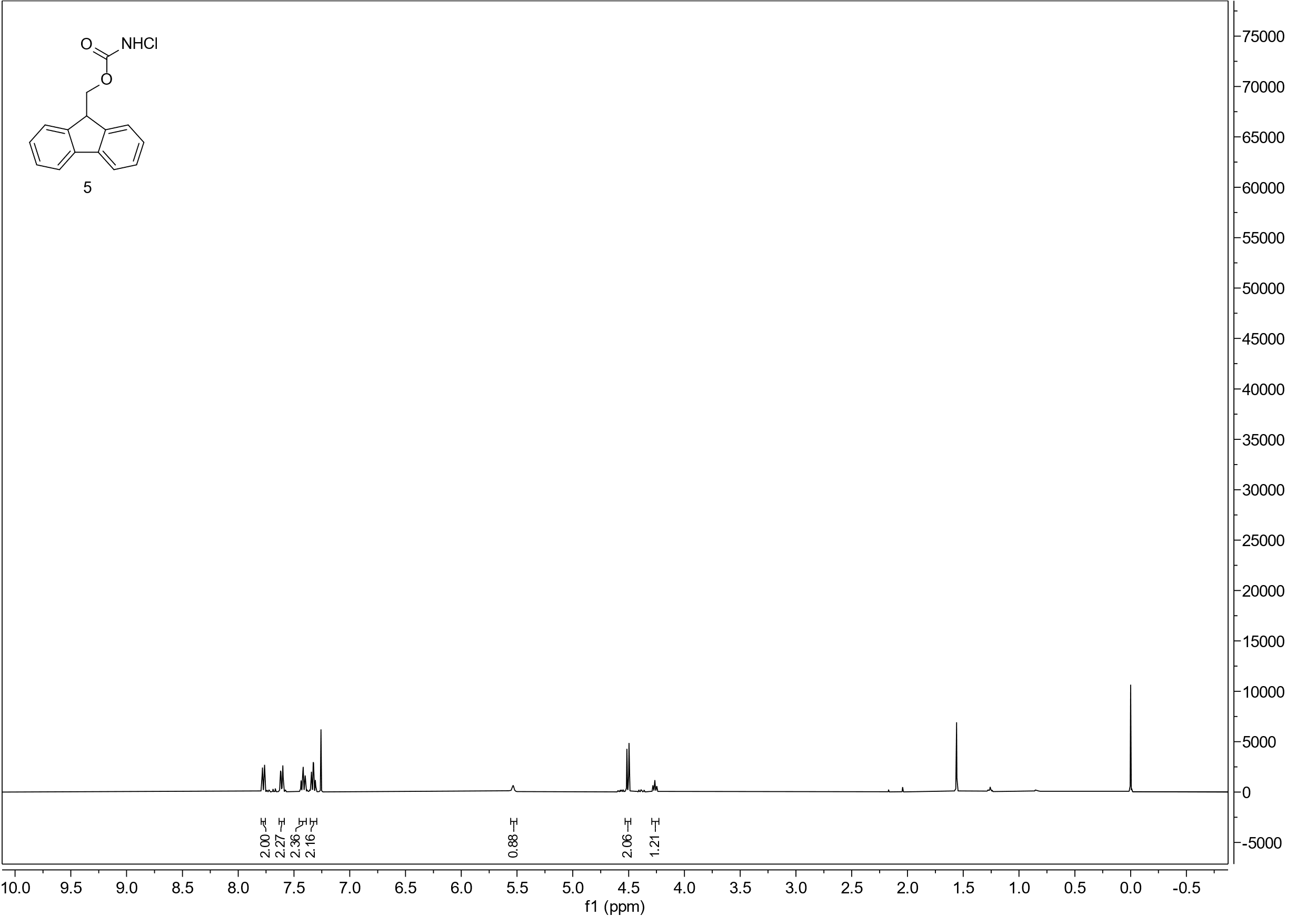


101 MHz, CDCl_3_


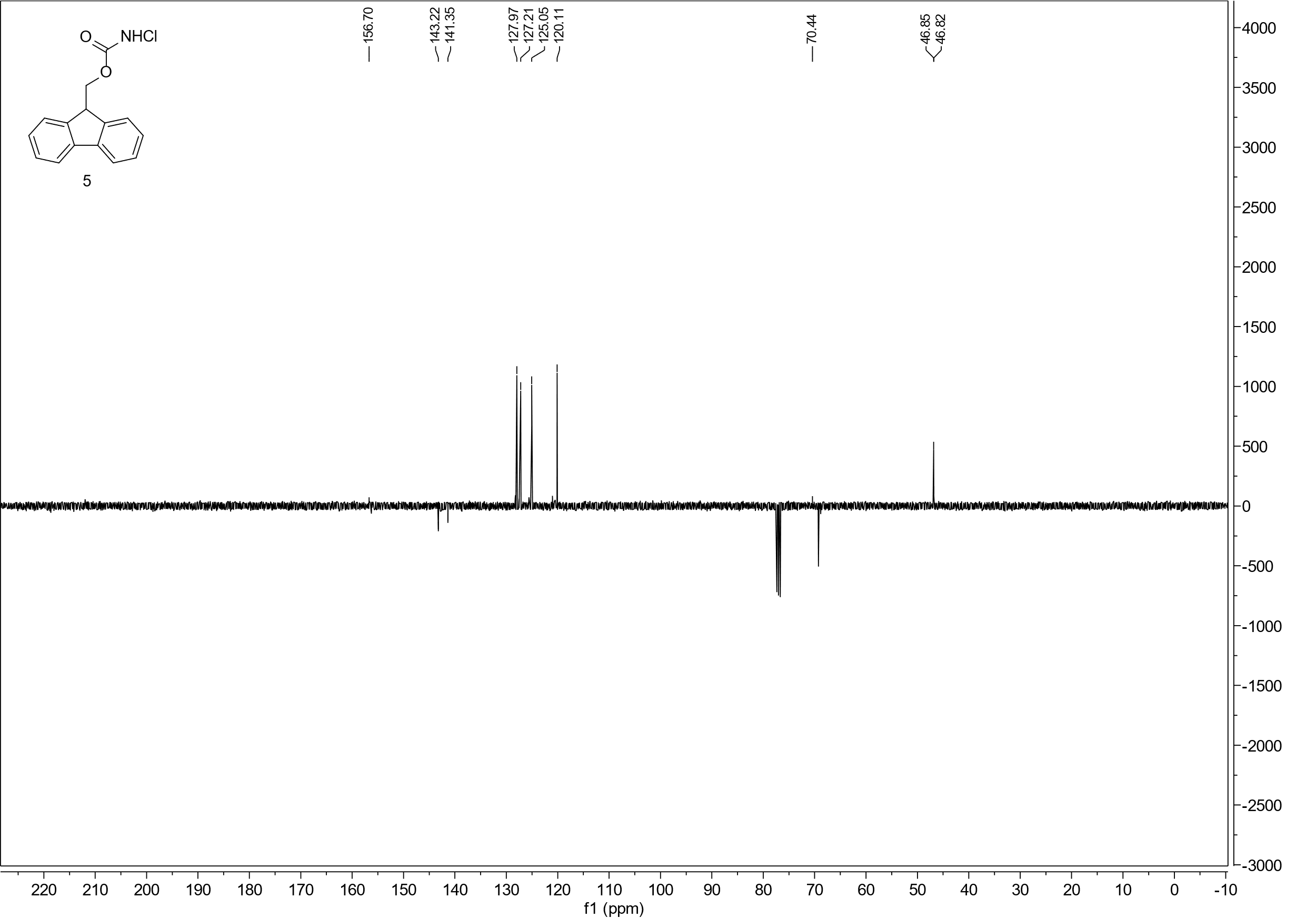


### **Supplementary Figure 26: NMR spectra of compound 2**

400 MHz, CDCl_3_


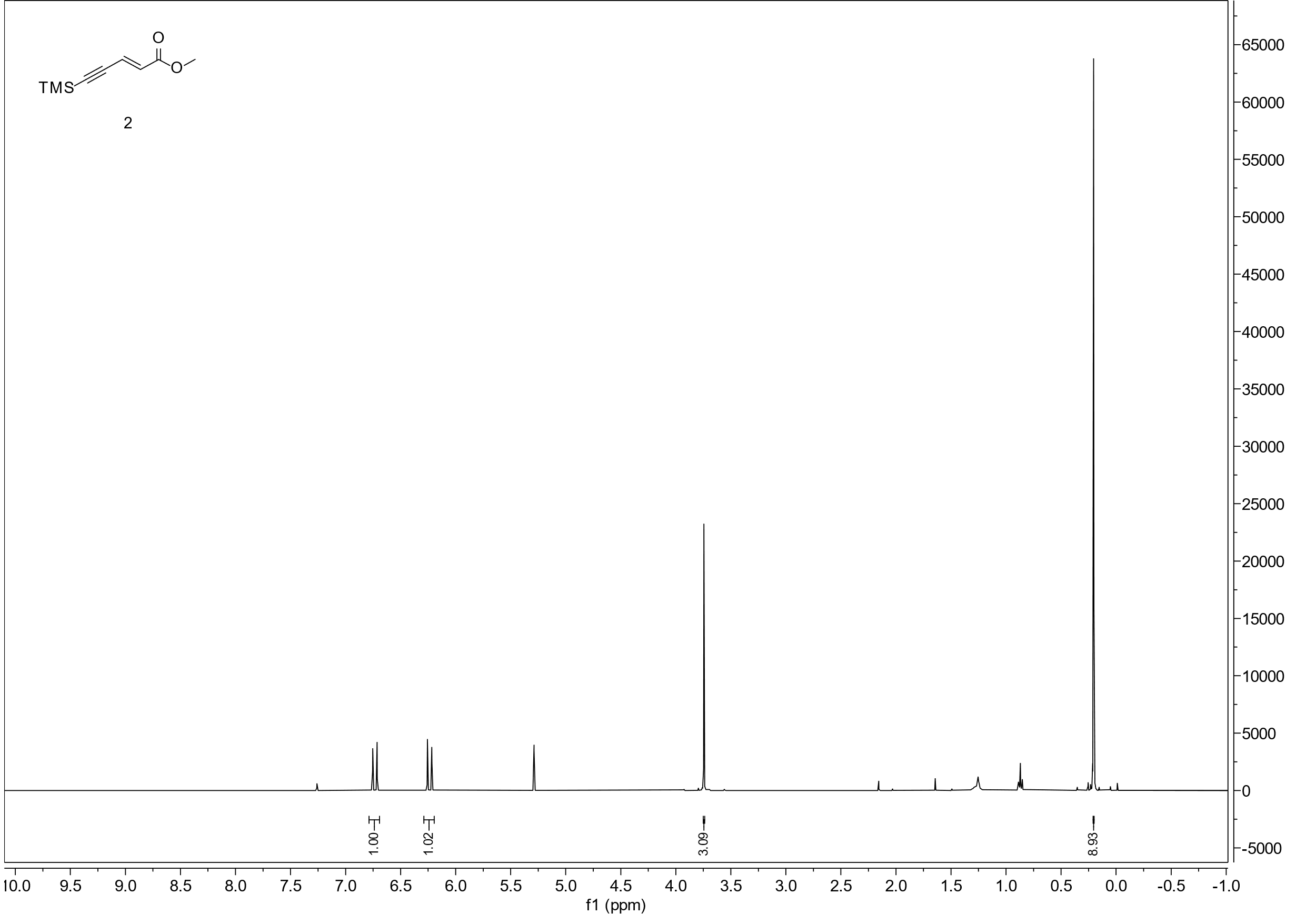


101 MHz, CDCl_3_


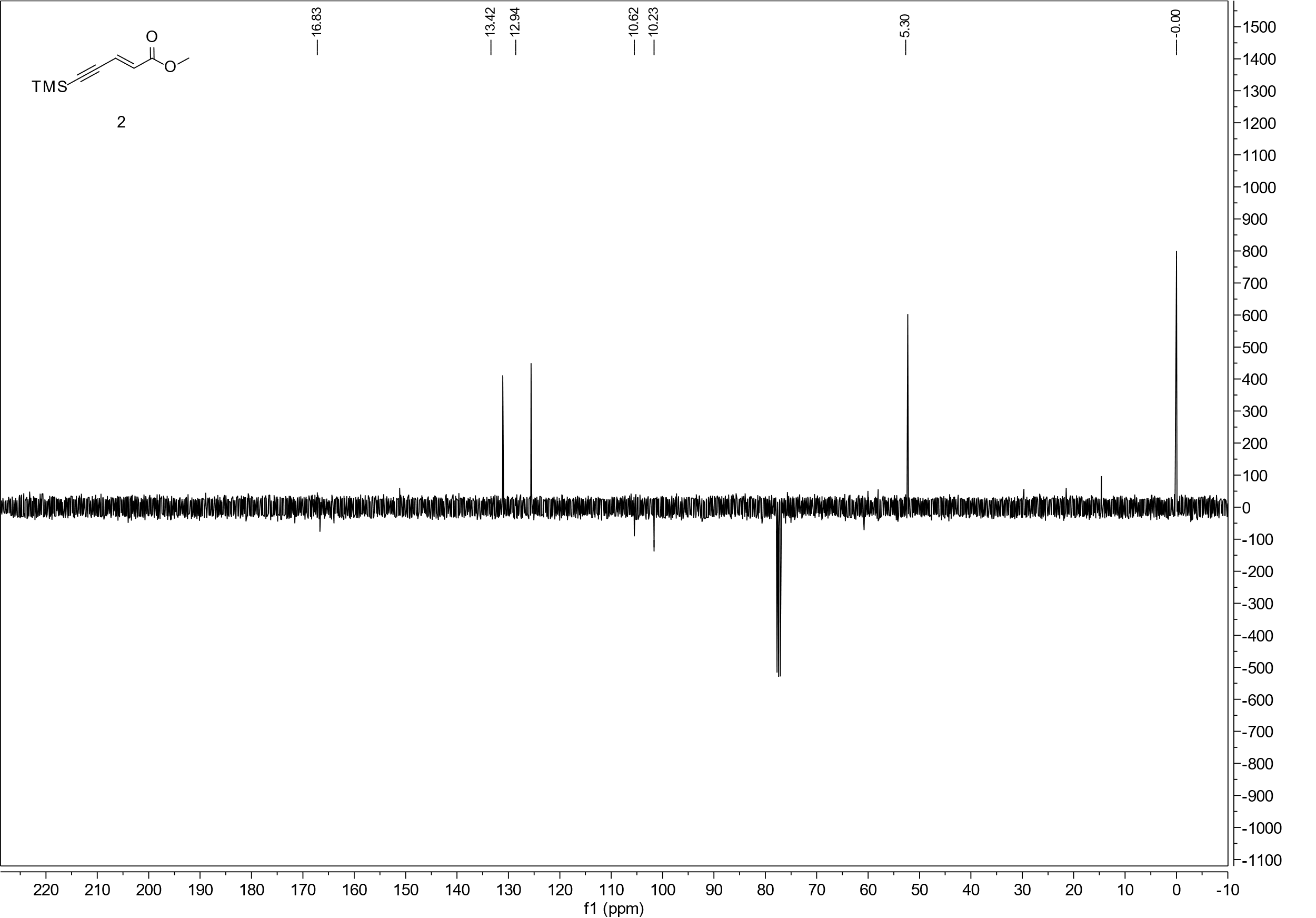


### **Supplementary Figure 27: NMR spectra of compound 3**

400 MHz, CDCl_3_


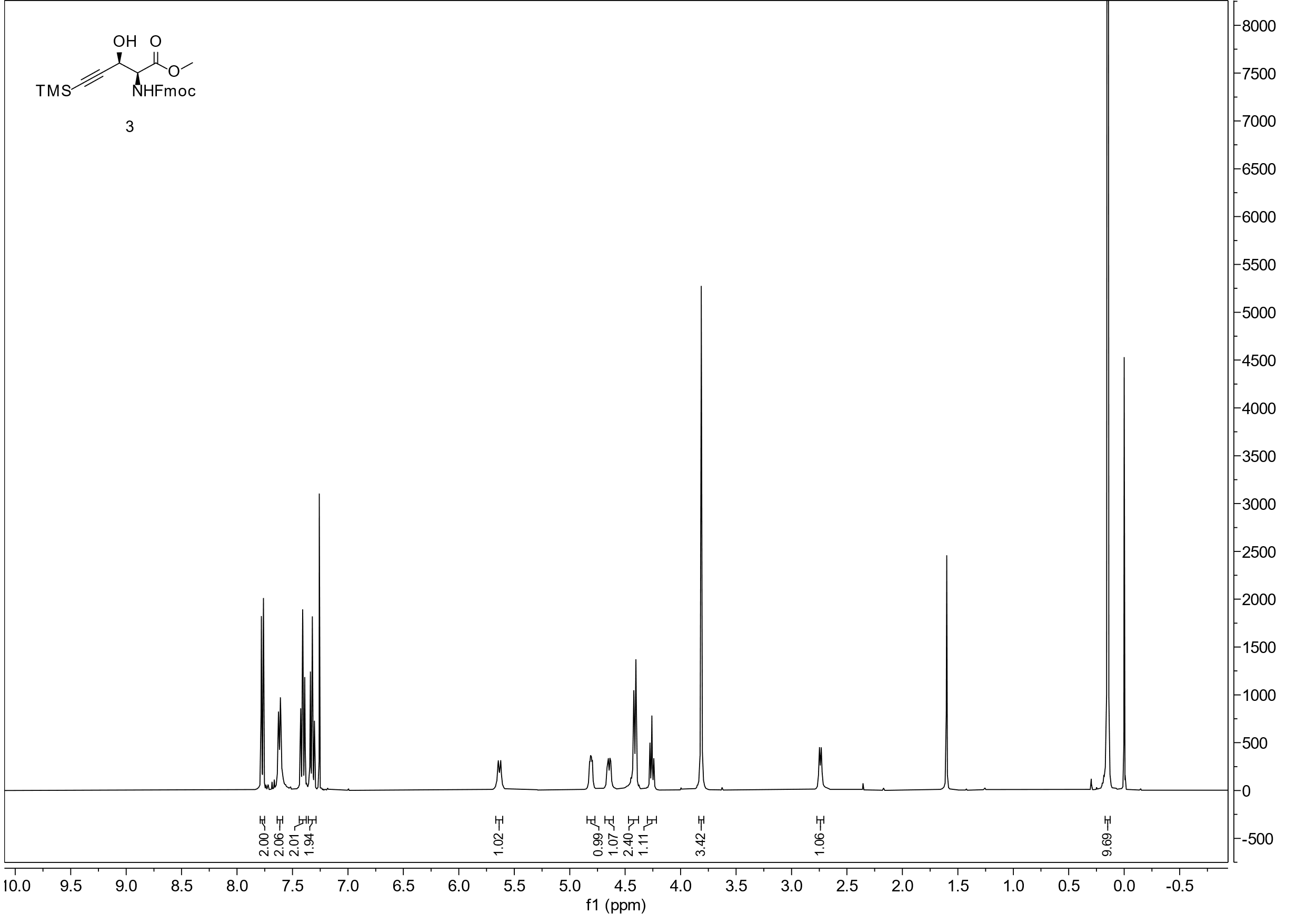


101 MHz, CDCl_3_


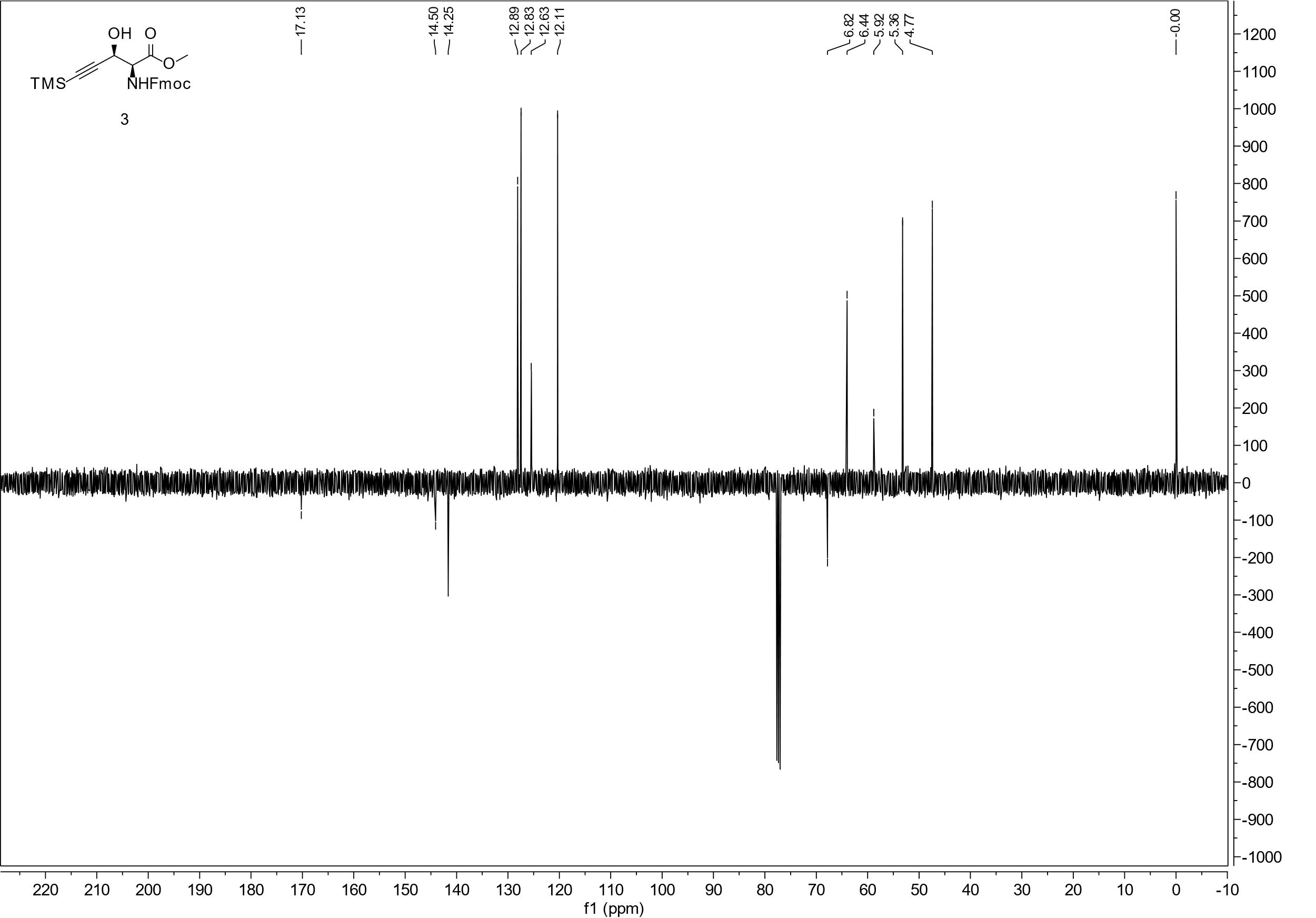


### **Supplementary Figure 28: NMR spectra of compound 1**

500 MHz, D_2_O

#
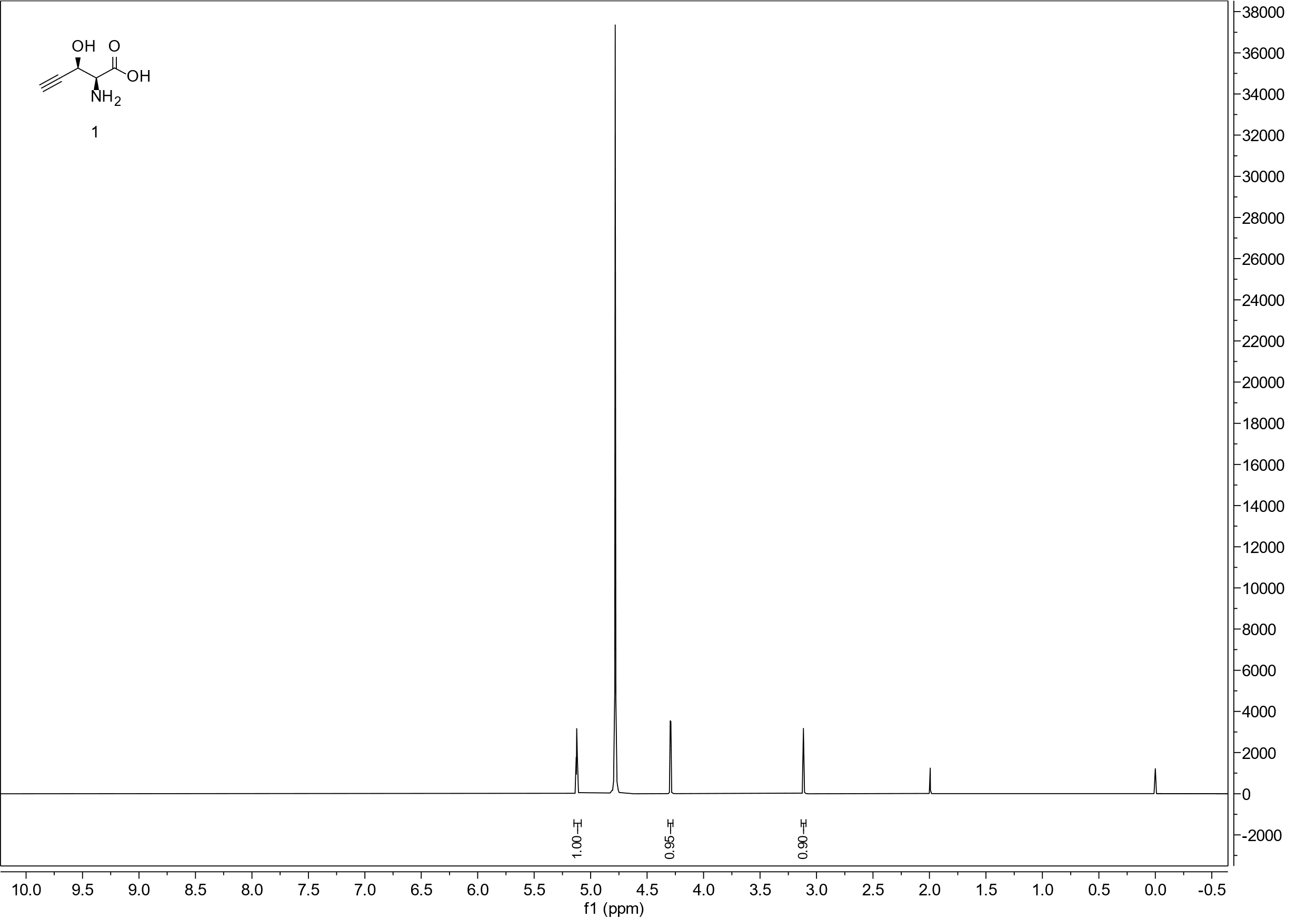


126 MHz, D_2_O


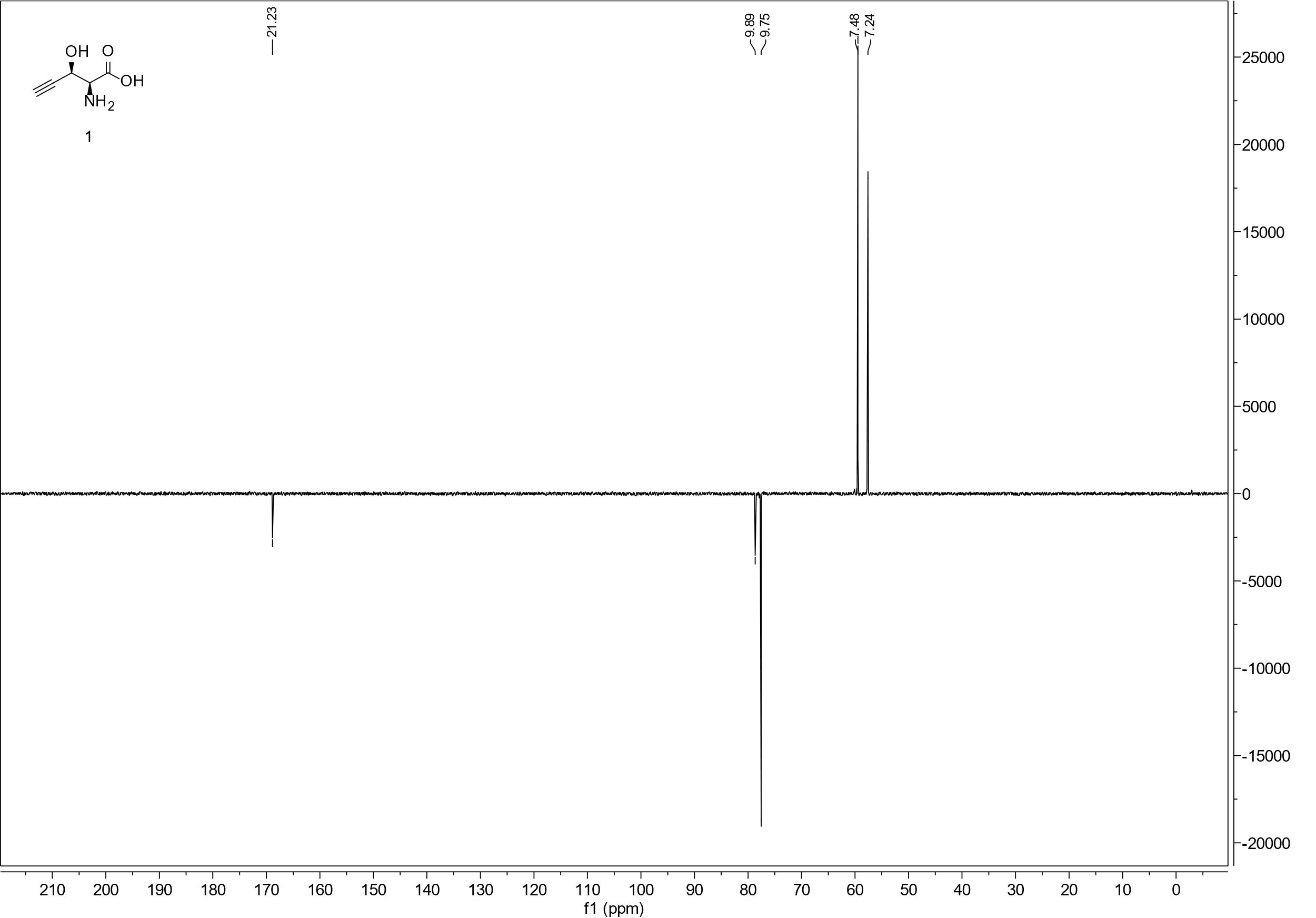


**Supplementary References**

1. Zecha, J. *et al.* Peptide Level Turnover Measurements Enable the Study of Proteoform Dynamics *. *Mol. Cell. Proteomics* **17**, 974–992 (2018).

2. Bagert, J. D. *et al.* Quantitative, Time-Resolved Proteomic Analysis by Combining Bioorthogonal Noncanonical Amino Acid Tagging and Pulsed Stable Isotope Labeling by Amino Acids in Cell Culture. *Mol. Cell. Proteomics* **13**, 1352–1358 (2014).

3. Niehues, S. *et al.* Impaired protein translation in Drosophila models for Charcot–Marie–Tooth neuropathy caused by mutant tRNA synthetases. *Nat. Commun.* **6**, 7520 (2015).

4. Moreira, R. & Taylor, S. D. Asymmetric Synthesis of Fmoc-Protected β-Hydroxy and β-Methoxy Amino Acids via a Sharpless Aminohydroxylation Reaction Using FmocNHCl. *Org. Lett.* **20**, 7717–7720 (2018).

5. Hoye, T. R., Jeffrey, C. S. & Shao, F. Mosher ester analysis for the determination of absolute configuration of stereogenic (chiral) carbinol carbons. *Nat. Protoc.* **2**, 2451–2458 (2007).
