# Supplementary material for: THRONCAT: metabolic labeling of newly synthesized proteins using a bioorthogonal threonine analog
Source: Nat Commun. 2023 Jun 8;14:3367. doi: 10.1038/s41467-023-39063-7 (PMC10250548; doi:10.1038/s41467-023-39063-7)
Supplement: Supplementary file 3 — Description of Additional Supplementary Files [file 41467_2023_39063_MOESM3_ESM.docx]

**Description of additional supplementary files**

**Supplementary Information**

Contains Supplementary Figures, Exact *p-*values for results in Fig. 5f (Supplementary Table 1), Custom media formulations (Supplementary Table 2), Supplementary Discussion 1, Organic syntheses and compound characterization (Supplementary Note 1) and Supplementary References.

**Supplementary Software**

Python code used to determine threonine- or methionine content of (sub-)proteomes.

**Supplementary Data 1**

Identified peptides and their intensities for βES- and Thr_5_-labeled HeLa cells.

**Supplementary Data 2**

Identified proteins and LFQ intensities for βES- and HPG-labeled HeLa cells.

**Supplementary Data 3**

List of proteins detected in HeLa cells by either THRONCAT or BONCAT, or both.

**Supplementary Data 4**

Identified proteins, LFQ intensities and statistical significance for βES-labeled Ramos cells.
