# Supplementary material for: THRONCAT: metabolic labeling of newly synthesized proteins using a bioorthogonal threonine analog
Source: Nat Commun. 2023 Jun 8;14:3367. doi: 10.1038/s41467-023-39063-7 (PMC10250548; doi:10.1038/s41467-023-39063-7)
Supplement: Supplementary file 9 — Reporting Summary [file 41467_2023_39063_MOESM9_ESM.pdf]

nature portfolio

Corresponding author(s): K.M.Bonger

Last updated by author(s): May 23, 2023

Reporting Summary

Nature Portfolio wishes to improve the reproducibility of the work that we publish. This form provides structure for consistency and transparency in reporting. For further information on Nature Portfolio policies, see our Editorial Policies and the Editorial Policy Checklist.

Statistics

For all statistical analyses, confirm that the following items are present in the figure legend, table legend, main text, or Methods section.

n/a	Confirmed	
☐	☒	The exact sample size (*n*) for each experimental group/condition, given as a discrete number and unit of measurement
☐	☒	A statement on whether measurements were taken from distinct samples or whether the same sample was measured repeatedly
☐	☒	The statistical test(s) used AND whether they are one- or two-sided<br>*Only common tests should be described solely by name; describe more complex techniques in the Methods section.*
☒	☐	A description of all covariates tested
☒	☐	A description of any assumptions or corrections, such as tests of normality and adjustment for multiple comparisons
☐	☒	A full description of the statistical parameters including central tendency (e.g. means) or other basic estimates (e.g. regression coefficient) AND variation (e.g. standard deviation) or associated estimates of uncertainty (e.g. confidence intervals)
☐	☒	For null hypothesis testing, the test statistic (e.g. *F*, *t*, *r*) with confidence intervals, effect sizes, degrees of freedom and *P* value noted<br>*Give P values as exact values whenever suitable.*
☒	☐	For Bayesian analysis, information on the choice of priors and Markov chain Monte Carlo settings
☒	☐	For hierarchical and complex designs, identification of the appropriate level for tests and full reporting of outcomes
☒	☐	Estimates of effect sizes (e.g. Cohen's *d*, Pearson's *r*), indicating how they were calculated

Our web collection on statistics for biologists contains articles on many of the points above.

Software and code

Policy information about availability of computer code

Data collection	Leica SP8 laser scanning confocal microscope was used for image acquisition of Drosophila immunofluorescence samples.<br>Leica MC170 HD microscope camera was used to collect images of Drosophila larvae.<br>LasX (Leica) was used for acquisition of immunofluorescence images.<br>Nikon D3100 DSLR camera was used for the recording of Drosophila climbing assays.<br>Leica DMi8 widefield microscope was used for image acquisition of HeLa fluorescence images.<br>BD Biosciences FACSVerse cell analyser was used for flow cytometry of HeLa cells.<br>ThermoScientific Orbitrap Exploris 480 was used for mass-spectrometry based proteomics data acquisition.<br>Bacterial growth curves were obtained on a Biotek 800TS plate reader.<br>In-gel fluorescence was measured on a Cytiva Typhoon 5 gel scanner.<br>Silver stain intensity for SDS-PAGE gels was measured on Bio-rad GelDoc XRS+ gel imager.
Data analysis	ImageJ 1.53 software (Sun Microsystems, USA) was used for analysis of microscopy images.<br>The movies of Drosophila climbing assays were converted into 8-bit grayscale TIF image sequences with 10 frames/s and analyzed using an MTrack3 plug-in that automatically imports images in ImageJ, subtracts backgrounds, and filters and binarizes images to allow tracking of flies.<br>For mass-spectrometry based proteomics experiments, MaxQuant v1.6.0.1 was used to identify and quantify proteins. Missing values were imputed using the R DEP package v1.14.0. R LIMMA package v3.48.3 was used to convert alias gene names to official gene names.<br>Overrepresentation analysis was performed with DAVID 2021.

Flow cytometry data was analyzed using FlowJo X v10.0.7r2.
Custom Python 3 script was used to determine threonine/methionine residue content in datasets (available as Supplementary Software)
All statistical analysis was performed using GraphPad Prism v.9.2.0.
SDS-PAGE gel lane intensity was measured using GelQuant.NET software (biochemlabsolutions.com)

For manuscripts utilizing custom algorithms or software that are central to the research but not yet described in published literature, software must be made available to editors and reviewers. We strongly encourage code deposition in a community repository (e.g. GitHub). See the Nature Portfolio guidelines for submitting code & software for further information.

Data

Policy information about availability of data

All manuscripts must include a data availability statement. This statement should provide the following information, where applicable:

- Accession codes, unique identifiers, or web links for publicly available datasets
- A description of any restrictions on data availability
- For clinical datasets or third party data, please ensure that the statement adheres to our policy

The mass spectrometry proteomics data have been deposited to the ProteomeXchange Consortium (http://proteomecentral.proteomexchange.org) via the PRIDE partner repository with the dataset identifier PXD032368. Uniprot reference proteome (UP000005640) was used as complete human proteome to analyze average proteomic threonine and methionine content.
The authors declare that all data supporting the findings of this study are available within the paper and its Supplementary Information files or from the corresponding author upon request.

Human research participants

Policy information about studies involving human research participants and Sex and Gender in Research.

Reporting on sex and gender	N/A
Population characteristics	N/A
Recruitment	N/A
Ethics oversight	N/A

Note that full information on the approval of the study protocol must also be provided in the manuscript.

Field-specific reporting

Please select the one below that is the best fit for your research. If you are not sure, read the appropriate sections before making your selection.

[x] Life sciences [ ] Behavioural & social sciences [ ] Ecological, evolutionary & environmental sciences

For a reference copy of the document with all sections, see nature.com/documents/nr-reporting-summary-flat.pdf

Life sciences study design

All studies must disclose on these points even when the disclosure is negative.

Sample size	For animal model studies, we used samples size within the typical sample size for similar studies see Zuko et al., 2022 Science<br>For all other experiments, n = 3 was used as minimal standard sample size allowing statistical testing, unless material availability allowed for larger samples sizes.
Data exclusions	No data were excluded.
Replication	For Drosophila, experiments were performed on the indicated number of animals, hence served as biological replicates.<br>For SDS-PAGE experiments, experiments were repeated at least twice with similar results. The exact number of replicates is indicated in the respective figure captions.<br>For mass-spectrometry based proteomics experiments, the number of biological or technical replicates is included in the respective figure captions.<br>All details on number of biological replicates are provided in the figure legends and supplementary data files.
Randomization	Allocation was random.
Blinding	For Drosophila, experimenters were blinded to the conditions and/or genotypes. In all other experiments , experimenters were not blinded as no bias was expected.

Reporting for specific materials, systems and methods

We require information from authors about some types of materials, experimental systems and methods used in many studies. Here, indicate whether each material, system or method listed is relevant to your study. If you are not sure if a list item applies to your research, read the appropriate section before selecting a response.

Materials & experimental systems

n/a	Involved in the study
☐	☒ Antibodies
☐	☒ Eukaryotic cell lines
☒	☐ Palaeontology and archaeology
☐	☒ Animals and other organisms
☒	☐ Clinical data
☒	☐ Dual use research of concern

Methods

n/a	Involved in the study
☒	☐ ChIP-seq
☐	☒ Flow cytometry
☒	☐ MRI-based neuroimaging

Antibodies

Antibodies used	anti-Discs large 1 (anti-dlg1; obtained from Developmental Studies Hybridoma Bank; clone name: 4F3; 1/200;)<br>anti-Brp (anti-nc82; obtained from Developmental Studies Hybridoma Bank; clone name: nc82; 1/100)<br>anti-IgG (Invitrogen, Cat. No. 31143)
Validation	These antibodies were used based on their large use in multiple studies as shown on manufacturer's websites:<br>-https://dshb.biology.uiowa.edu/4F3-anti-discs-large<br>-https://dshb.biology.uiowa.edu/nc82

Eukaryotic cell lines

Policy information about cell lines and Sex and Gender in Research

Cell line source(s)	HeLa cells (ATCC)<br>Ramos 3F3 cells (obtained from prof. R.E.M. Toes, LUMC, Netherlands; for details see Kissel et al., 2020 Ann. Rheum. Dis.)
Authentication	Cell lines were not authenticated.
Mycoplasma contamination	Cells were negative for mycoplasm
Commonly misidentified lines (See ICLAC register)	No commonly misidentified lines used in this study

Animals and other research organisms

Policy information about studies involving animals; ARRIVE guidelines recommended for reporting animal research, and Sex and Gender in Research

Laboratory animals	Drosophila lines:<br>-OK371-GAL4, UAS-mCD8-GFP;UAS-mCD8-GFP was kindly provided by M.Freeman<br>-W1118 was obtained from Bloomington Stock Center (BL 3605)<br>-UAS-GARS-G240R is a ESt lab strain that was previously described in Niehues et al., 2015, Nat. Commun.<br>-OK371-GAL4> UAS-MetRSL262G is a ESt lab strain that was previously described in Erdmann et al., 2015, Nat. Commun.<br>In all experiments third instar larvae were used except Supplementary Figure 20F where 4-5 day old flies were studied and Supplementary Figure 22 where third instar larvae were followed over time until 1 day old flies.
Wild animals	no wild animals were used
Reporting on sex	In Drosophila experiments the information on sex was not collected.
Field-collected samples	no field collected samples were used
Ethics oversight	No ethical oversight required.

Note that full information on the approval of the study protocol must also be provided in the manuscript.

Flow Cytometry

Plots

Confirm that:

- [x] The axis labels state the marker and fluorochrome used (e.g. CD4-FITC).
- [x] The axis scales are clearly visible. Include numbers along axes only for bottom left plot of group (a 'group' is an analysis of identical markers).
- [x] All plots are contour plots with outliers or pseudocolor plots.
- [x] A numerical value for number of cells or percentage (with statistics) is provided.

Methodology

Sample preparation	In all cases, input cells came from stable cell lines and were fixed using paraformaldehyde before flow cytometry analysis.
Instrument	BD Biosciences FACSVerse
Software	FlowJo X 10.0.7r2
Cell population abundance	A minimum number of n = 10.000 events were measured (before gating) for each replicate.
Gating strategy	For all flow cytometry, the following gating strategy was applied:<br>- FSC-A vs. SSC-A wide gate excluding only small debries<br>- FSC-A vs. FSC-W tight gate around main cell population<br>- FSC-A vs. SSC-H tight gate round main population<br>The final child population was used to determine mean fluorescent intensity (MFI).

- [x] Tick this box to confirm that a figure exemplifying the gating strategy is provided in the Supplementary Information.
